# Without Assumptions: Development of a Socio-Emotional Learning Framework That Reflects Community Values in Cameroon

**DOI:** 10.3389/fpubh.2021.602546

**Published:** 2021-05-07

**Authors:** Brigitte Anziom, Sarah Strader, Anselme Simeon Sanou, Philip Chew

**Affiliations:** ^1^L'Association pour la Traduction, l'Alphabétisation, et le Développement Holistique de l'Etre Humain (ASTRADHE), Lomié, Cameroon; ^2^Two Rabbits, Washington, DC, United States; ^3^Centre Muraz Research Institute, Bobo Dioulasso, Burkina Faso; ^4^Independent Consultant, Cambridge, MA, United States

**Keywords:** socioemotional learning, early childhood development, indigenous education, Cameroon, Baka (Pygmies), assessments, community-based education, rapid ethnographic assessment

## Abstract

Socioemotional learning (SEL) skills are the competencies that children need to be successful and accepted members of society. In this study, we built a SEL framework and a SEL measurement tool from the ground up that assess children's development of skills with communities of the Baka ethnic group in Cameroon. We conducted a participatory and interactive study to develop a SEL framework and measurement tool that is specific to the context of indigenous Baka communities in Cameroon. Using a quick ethnography methodology and an emic approach, a researcher team comprised mainly of Baka community members engaged parents, teachers, and others in iterative cycles of data collection, analysis, and reflection to develop the framework and assessments. The resulting Baka SEL framework includes skills and domains distinct from predominant SEL frameworks, underscoring the importance of drawing SEL priorities from communities themselves. Shared foundational constructs underlying the Baka SEL framework and other frameworks indicate possible universal human expectations for emotional and relational skills. Two SEL measurement tools were produced: a caregiver tool and a teacher tool, each using storytelling to elicit specific, honest, and detailed information about child behavior. These tools allow us to capture child behavior in the school and the home, and to collect data on all participating children within a specific time period. The described approach is a simple, practical, and culturally appropriate strategy for collaborating with rural communities to articulate their understanding of SEL. The resulting framework and tools illustrate the importance of rooting SEL in local culture, while the approach to developing them serves as a model for other early childhood care and education organizations and programs.

## Introduction

Numerous studies have demonstrated the importance of increased socioemotional learning (SEL) skills in improved quality of life into adulthood, and that preschool programs can help build these skills (1–5). Despite increasing popularity of SEL programs and measures, research supporting the underlying psychological frameworks is lacking, resulting in controversy and inconsistency in the definition, measurement, and utility of SEL (6). SEL skills are closely linked to societal values, comprising the necessary attributes to succeed in a specific culture and environment (7, 8). Anthropological studies find that these attributes vary from one context to another, and existing SEL frameworks do not apply uniformly across individuals, groups, systems, or—especially—different cultural contexts (1, 9–11). Early childhood care and education programs that develop SEL skills must ensure their work is rooted in a localized understanding of SEL.

Predominant approaches for child development measurement in international contexts, including measurement of SEL, entails the adaptation of existing measures to new settings (12–15). Humphrey et al.'s (8) systematic review of existing SEL measures finds the majority of these tools were developed and standardized with American populations. Research has concluded that certain measures can be translated and adapted to different cultures while retaining acceptable reliability and validity. However, cross-cultural and context-specific norms are seldom provided in these measures or validity studies (8). Retrofitting a tool developed for children in a certain cultural context to a historically marginalized society risks carrying over assumptions about valuable skills from one culture to another. While the literature is clear that defining and measuring SEL with measures that bear psychometric muster for formative purposes is useful, missing from the literature is an empirical evaluation of a SEL measure that is first defined and constructed within an indigenous context.

Organized formal schooling in indigenous communities worldwide has historically served as a colonizing force that extracts children from their culture and imposes another (16). The present study took place in indigenous Baka communities in Cameroon. Baka children experience stigma in school, which contributes to feelings of shame about their culture (17–19). Because few Baka children speak French at home, and daily classroom learning requires children to miss foraging activities, Baka children drop out of school at higher rates than non-Baka peers. A 2018 survey found that 80% of Baka adults identify as illiterate (20). A Baka father stated that the Baka need to “chase two rabbits at once” by pursuing both ancestral forest-based education and formal schooling (21). Chasing two rabbits entails learning multiple sets of affective and interpersonal skills, as well as applying them in different settings (a practice often referred to as code switching), in order to garner respect and achieve their objectives within each setting (22, 23). Schools must actively counter structural discrimination that often leads children to view the dominant society, skill set, or culture as superior to their ancestral one. Research indicates that schools welcoming indigenous children must have teachers, leaders, and curriculum that value children's experiences and identities, and ensure indigenous presence in the school through active engagement of caregivers (22, 24–27).

In order to understand the SEL skills that Baka children need for success, we conducted a participatory study to answer two research questions: (1) How do Baka caregivers conceptualize SEL skills that children need for success? (2) How can a measurement tool faithfully and validly capture progress in developing these skills in early childhood? The objectives of this study are therefore (1) to build a Baka SEL framework and (2) to build a Baka SEL measurement tool that assesses children's development of skills in the Baka SEL framework.

The study describes a participatory and user-friendly procedure for engaging communities meaningfully to achieve these two goals. While SEL frameworks and measures often target problem behaviors, this study seeks to understand and track children's positive development as they learn to “chase two rabbits at once.” As our key objective was to ensure fidelity to community values and SEL priorities, conceptual and face validity are critical factors in the analysis of study results.

## Materials and Methods

### Study Area and Setting

The Baka are an indigenous ethnic group in the East and South Regions of Cameroon. They are historically a semi-nomadic forager society, with primary livelihood activities including hunting, fishing, and gathering. Over the past 50 years, efforts by government, religious, and civil society organizations have sought to transition them to sedentary agriculture and animal husbandry. There has been a simultaneous increase in activities that significantly limit Baka access to forest resources, including logging, poaching, mining, and creation of wildlife conservation zones. This uprooting has coincided with increases in alcoholism, depression, and malnutrition in Baka villages.

The study is implemented in the context of Dεngbε Bide, a community-based preschool initiative. The Dεngbε Bide model targets building children's pride in their home culture through their first experiences in a classroom setting. The mission of Dεngbε Bide is to support Baka children to pursue their traditional forest-based education, while also preparing them for success in formal primary school. Dεngbε Bide partners with Baka communities to create community preschool centers (CPC) by developing interactive audio curricula rooted in the Baka language and environment, and training community-nominated youth to serve as teachers.

### Qualitative Approach and Research Paradigm

With a view to preparing children to “chase two rabbits at once,” it was critical to map and capture SEL skills essential for success in Baka society in particular. Rather than starting with an existing tool for adaptation, the research team opted to develop a tool from the ground up through a participatory and interactive process that engaged caregivers as co-investigators.

The study design reflects principles of rapid ethnography. Rapid ethnography was created as a simple and practical methodology for project teams to answer “how” and “what” questions by gathering evidence iteratively in partnership with communities (rather than the researchers positioning themselves as experts) (28). As in the Dεngbε Bide program itself, expertise flows from local individuals immersed in their natural setting (28, 29). Consistent with the research team's commitment to proceed “without assumptions,” rapid ethnography methodologies embrace an emic approach to data collection and analysis whereby observations from community members serve as “the foundation for understanding” (30). We collected data through an iterative series of interviews with open-ended questions, prioritizing both broad participation and deep community engagement in building common understandings of SEL (29–32). We used an iterative process with multiple rounds of data collection and analysis, in order to regularly revisit findings and adjust our protocols. Consistent with rapid appraisal methods, instead of seeking to prove or disprove a research hypothesis, we used data to develop, clarify, and revise hypotheses through successive rounds of collection, analysis, and reflection.

To achieve our first study objective, we entered the study without a preconceived framework, instead building the Baka SEL framework from the ground up with caregivers. For our second study objective, we created questionnaires based on the Baka SEL framework, and engaged community members in multiple rounds of tool testing to develop and refine its items and administration procedures. While the study used an inductive paradigm, its outcome was to develop a deductive tool that could be used to objectively assess SEL development. Similarly, the study involved qualitative data collection and analysis, and used findings to develop a SEL measure that employs qualitative questioning to arrive at a quantitative evaluation of SEL skill mastery.

### Research Team

The core research team included five ASTRADHE staff members: three Baka men, one non-Baka man, and one non-Baka woman from predominantly Baka villages. They were supported by a female American specialist in early childhood development. This composition was designed to include diverse backgrounds and disciplines, with a mix of community “insiders” and “outsiders,” consistent with recommended practice in rapid ethnography (33, 34). The core team was supported by an extended community team, comprised of caregivers within the Baka community. Table 1 summarizes each team's composition and responsibilities.

**Table 1:** Research team composition and responsibilities.

	**Core team**			**Extended community team**		
Role	Propose and refine data collection tools and procedures Develop data analysis procedures Co-analyze data, clarify findings Develop and pilot questionnaires			Review and recommend revisions to data collection tools and procedures Co-analyze data, clarify findings Review and suggest updates to questionnaires		
Composition		Men	Women		Men	Women
	5–10 yrs schooling	2 age 40+ 2 age 18–40		0–3 yrs schooling	3 age 40+ 3 age 18–40	3 age 40+ 3 age 18–40
	10+ yrs schooling		1 age 40+ 1 age 18–40	3+ yrs schooling	3 age 40+ 3 age 18–40	3 age 40+ 3 age 18–40

### Data Collection and Analysis Methods

Our methodology is presented in two phases. In phase one, we developed the Baka SEL framework. This framework informed phase two of developing a Baka SEL measurement tool. However, these phases were not perfectly sequential, as learnings from phase two also allowed us to further refine the Baka SEL framework developed in phase one. Our process of data collection, reflection, follow-up, refinement of questions, and development of conclusions is described below. Data are available upon request via email to the correspondence author Strader.

#### Phase 1: Develop Baka SEL Framework

To develop the Baka SEL framework, we established internal consensus on the meaning of SEL, developed and administered open-ended questionnaires to Baka caregivers, conducted qualitative open-coding of their responses into SEL domains and skills, and integrated apparently salient skills from the global SEL literature. We compiled this information for community input and updated the tool to incorporate their feedback to create the final Baka SEL framework.

##### Step 1: Establish a Common Internal Definition of SEL

The core research team began with a review of popular SEL frameworks, in order to establish common internal understanding of how SEL is defined, how frameworks are constructed in other contexts, and strategies to ascertain caregiver views on SEL without influencing their responses. In reviewing the literature, we found that there is a lack of a consensus among researchers around a common neutral definition of SEL; explanations of SEL tend to take the form of a list of SEL skills or domains. For example, Yates et al. (35) define SEL as the ability to “form close and secure adult and peer relationships; experience, regulate, and express emotions in socially and culturally appropriate ways; and explore the environment and learn” [in (1)]. This complicated the task of seeking caregivers' conceptions of SEL skills, as we needed to formulate the question without providing a partial answer. The core team sought to address this by focusing on the importance of SEL skills by reflecting on the question: what does mastery of these skills result in, and allow children to achieve? The team identified achieving personal goals and community respect as key outcomes of SEL development, and as neutral starting points for designing interview protocols to engage caregivers in discussion about what SEL means to them.

##### Step 2: Develop and Implement Caregiver Interview Protocol

We designed a questioning strategy to draw out caregiver views on the skills that children need to succeed in Baka society. To do so without offering preconceived definitions or examples, we employed a “quick ethnography” method based on a study in Afghanistan to create a localized measure of mental health (29, 31). This approach uses storytelling and open coding to build the construct from the ground up. Our objective was to have caregivers describe children who are capable and respected in the community, and children who are not. Our intention was to obtain descriptions of children who exemplify the skills and attributes to be a positive member of Baka society, and children whose behavior contradicts societal expectations around these positive skills and attributes. These data would yield observable and specific attributes at both “ends of the spectrum” of SEL skills Baka caregivers feel are important. The core team developed an initial questionnaire and engaged four members of the extended community team (one older man and woman, and one younger man and woman) to pilot test it. Testing entailed three steps. First, the core team posed a question. Second, extended community team members responded to the questionnaire items, and then rephrased the question in their own words. Finally, core team members revised and re-posed the question until there was a common understanding of the item. After two rounds of revision, the core team arrived at wording that was consistently understood as intended—as summarized in Table 2—and proceeded to administer the questionnaire to the extended community team.

**Table 2:** Caregiver open questionnaire.

**Caregiver Questionnaire**
1. Think of a specific child that you know between the ages of 3 and 5 that has a “good head” and gets along well with others.^a^ a. What does this child do that shows you that he/she has a “good head” and gets along well with others? b. How did this child learn these behaviors? 2. Think of a specific child that you know between the ages of 3 and 5 that is a bit of a “head smacker,” and does not get along well with others. a. What does this child do that shows you that he/she is a “head smacker” and does not get along well with others? b. How did this child learn these behaviors?

a *This is a direct translation from Baka. Testing revealed that parents found the question most clear when we described children in this way. A child with a “good head” is one who generally embodies community values and is admired by adults. A “head smacker” can be understood as a child whose behavior makes you slap your forehead and wonder how he or she got that way*.

##### Step 3: Analyze Caregiver Interviews to Identify SEL Domains and Indicators

**Figure 1:**
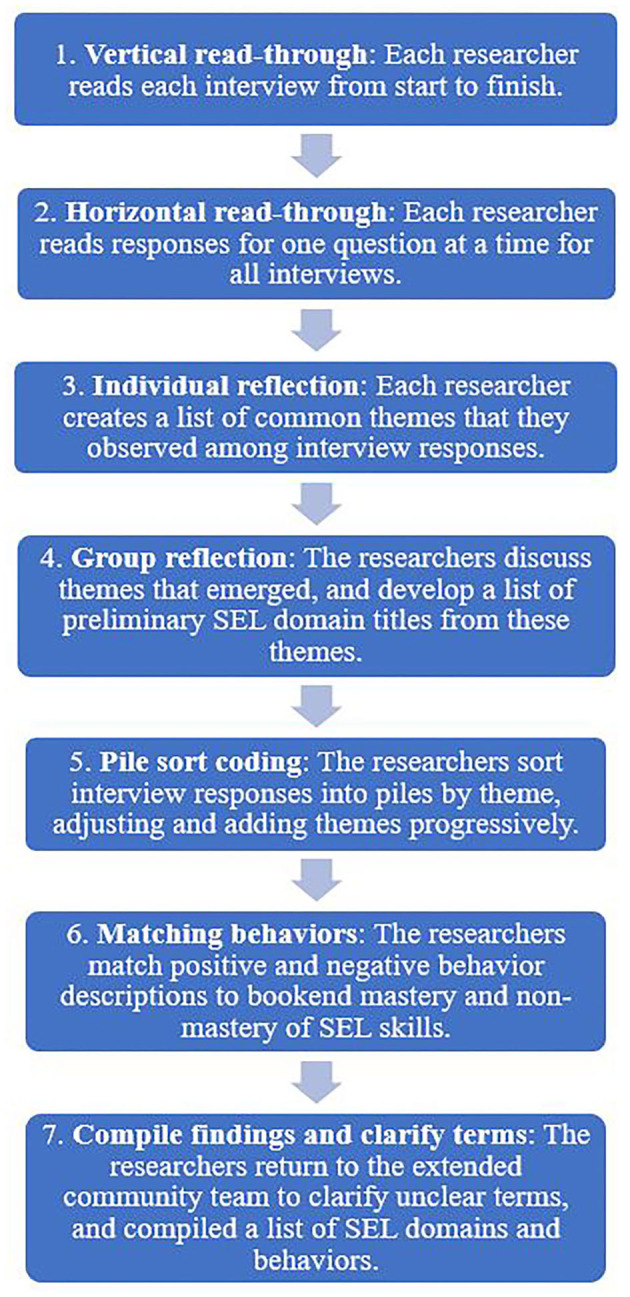
Caregiver interview analysis procedure.

Our next task was to cull data resulting from step 2 to identify themes that flow directly from caregivers' testimonies. The objectives of this step were to identify SEL skills as Baka communities perceive them, organize them into domains, and detect indicators of child behaviors at different levels of mastery for each skill. Given our team's remote location and our desire for replicability in similar contexts, our process sought to enable the group to collaboratively analyze a large amount of qualitative data, without the use of any software. Our approach is summarized in Figure 1.

After reading interview transcriptions twice and noting themes they observed, the team discussed and compiled preliminary domains into a single list. We then conducted a pile sort of caregiver responses. We formed one pile for each preliminary SEL domain on a table. We printed transcribed caregiver responses and cut them into strips of paper, with one sentence per strip. Reading each aloud one by one, we placed each strip in the SEL domain pile that appeared to fit best. Throughout the process, we iteratively adjusted the wording of domains, created new domains, and consolidated domains based on the content of the caregiver responses. This exercise resulted in a set of SEL domains (the piles), with series of indicators describing positive and negative behaviors pertaining to that domain (the strips). We then matched strips with positive and negative descriptions of similar behavior (such as a sharing or not sharing food), in order to identify behaviors that demonstrate mastery or non-mastery of SEL skills. Finally, the research team reviewed the sentences in the pile, created a title and one-sentence description for each domain, and compiled a list of behavior indicators from caregiver quotes on the strips that describe positive and negative behaviors that fall within that domain.

##### Step 4: Compare Baka SEL Framework With Other Global SEL Frameworks

We revisited the literature on internationally validated SEL measures (13, 36–38) to examine commonalities and differences in the SEL skills and behaviors included. This review served two purposes. Firstly, we identified through consensus SEL items and domains that appeared salient or obvious but did not figure strongly in caregiver responses, and integrated these into the framework for caregiver feedback on their relevance. Secondly, we explored skills and domains that are shared across frameworks and those that appear unique to the Baka SEL framework. This analysis sheds light on skills that may be universally salient, and how their manifestations may differ across cultures. It also highlights skills critical for success in Baka society that are absent from global frameworks, underscoring the importance of diverse cultural perspectives in defining SEL. Comparison of the Baka SEL framework with global SEL frameworks is presented in the Discussion section of this paper.

##### Step 5: Clarify Terms and Constructs

We returned to the extended community team to gain feedback on unclear terms from interviews, and proposed items drawn from the literature. During the caregiver interview analysis, we found that some caregivers described behaviors in specific and observable terms, while others spoke more generally. Some responses included behaviors and terms with multiple possible interpretations or unclear phrasing. They also included outlier data points, such as positive or negative behavior that only one caregiver raised, or which diverged from other caregivers' remarks. The core team went back to the extended community team to clarify these points, and to ascertain the saliency of skills and domains drawn from global SEL frameworks, by asking them to recount a story of the child who demonstrates a named behavior, and how they feel about a child that behaves in this way. The core team updated the domain titles, domain descriptions, and lists of behavior indicators based on caregiver responses. We then translated the titles, descriptions, and indicators into French and English.

#### Phase 2: Build Baka SEL Measurement Tools

To develop the Baka SEL measurement tools, we converted the Baka SEL framework into a questionnaire, conducted a small pilot for clarity of interpretation, and conducted a full pilot to analyze its psychometric properties. At each round of piloting, we integrated community remarks and lessons learned to strengthen its validity, reliability, and consistency.

##### Step 1: Develop Initial Questionnaire

To develop the items of the questionnaire, the core research team converted indicators of SEL skills from the Baka SEL framework into target behavior statements that describe mastery of each SEL skill, and non-mastery statements that describe lack of each skill. These statements came from two sources: caregiver quotes, and the existing SEL tools from which we sourced additional salient SEL skills.

In developing the questionnaire protocol, the team sought to maximize construct validity by minimizing influence on caregiver responses. During interviews in phase one, the research team found caregivers were reluctant to speak negatively of their own children, but were willing to describe negative behaviors of other children without saying their names. As the intention of the Baka SEL measurement tool is to obtain caregiver report of their own child's behavior, this tendency introduced a risk of bias. To identify strategies to mitigate this risk, the research team reflected on strategies from phase 1 that elicited honest and varied caregiver responses, and reviewed existing SEL measures validated in African contexts. We designed pilot questionnaire administration and scoring guidelines based on these learnings, and updated them through successive rounds of piloting.

In identifying respondents, the research team sought to obtain perspectives on child behavior in home and school settings, to assess each child in the Dεngbε Bide program, and to collect data at multiple time points to track progress in skill development. We theorized that targeting home caregivers (e.g., parents and extended family members) and Dεngbε Bide preschool teachers would achieve all three objectives. First, interviewing both groups would allow us to understand and compare adult perceptions of child behavior in classroom and home settings. Research and lived experience indicate that children behave differently in school and home environments, driven by different relationships and expectations of teachers and home caregivers (10). Dεngbε Bide teachers operate in community preschools that incorporate elements of a Baka home and a classroom. Children live by Baka values while also becoming acquainted with some expectations of formal schooling, such as following detailed explicit instructions, working on abstract problems, and working toward individual (rather than collective) results. Second, Baka home caregivers follow the Baka semi-nomadic rhythm of foraging activities, meaning that they are not regularly available in the village for interviews. Exclusively targeting caregivers would risk a biased sample of children whose families are less likely to be in the forest. Third, teachers are able to speak to behaviors of an entire class of children, while caregivers can speak only to their own child's behavior. Interviewing teachers allows for assessment of all preschoolers at the beginning and the end of the school year. Interviewing caregivers continuously during monthly program monitoring visits generates a continuum of data over multiple time points on child behavior at home.

##### Step 2: Conduct Initial Piloting and Revision

The core research team conducted two rounds of tool piloting and revision. After each round of piloting, we compiled findings and integrated improvements into each tool that are summarized in the results section below. Initial piloting sought to ascertain the salience of the SEL skills and domains, the clarity and consistency of question interpretation, and the feasibility and reliability of our scoring procedures. The initial piloting took place with five caregivers and two teachers, and the procedure is described in Table 3 below.

**Table 3:** Initial tool piloting procedure.

**For home caregivers**	**For teachers**
1) The assessor asks the open-ended question to the caregiver and asks the caregiver to restate the question in his or her own words. 2) If the caregiver's response does not correspond with the intention of the question, the assessor rephrases the question until the caregiver understands the meaning, and writes down the new phrasing. 3) The assessor asks the caregiver to respond to the question, and scores his or her response. 4) The assessor reads the target behavior statement aloud, asks the caregiver to give an example of the behavior they believe the target behavior statement refers to and writes down the response. 5) If the caregiver's example does not correspond with the intention of the target behavior statement, the assessor rephrases the question until the caregiver understands the meaning, and writes down the new phrasing. 6) At the end, the assessor asks the caregiver to note which questions are the hardest to understand, which behaviors are the most important for a child to have, and which behaviors were not so important for the child to have.	1) The assessor reads the target behavior statement aloud, and asks the teacher to restate the behavior in his or her own words. 2) If the teacher's response does not correspond with the intention of the question, the assessor rephrases the question until the teacher understands the meaning, and writes down the new phrasing. 3) The assessor asks the teacher to respond to the question, and scores his or her response. 4) At the end, the assessor asks the teacher to note which questions are the hardest to understand, which behaviors are the most important for a child to have, and which behaviors were not so important for the child to have.

##### Step 3: Conduct Full Tool Piloting, Psychometric Analysis, and Revision

The second round of piloting entailed assessing the tools' psychometric properties, to ascertain their ability to capture differences in child SEL development and identify opportunities to strengthen them. We administered the tool to 30 teacher pairs and 29 caregivers, gathering data on a total of 539 children. We used Stata 16.0 MP to assess the tool's score distributions, interclass correlation, internal consistency, inter-rater reliability, existence of latent factors, item test correlations, and assessor rating patterns. We also measured interclass correlation for the teacher tool, and inter-rater reliability for the caregiver tool. Finally, we analyzed the statistical relationship between caregiver and teacher ratings of the same child for each behavior. These findings served as the basis for improvements to the tools' structure, wording, and administration and scoring protocols for greater reliability and consistency.

## Results

### Building a Baka SEL Framework

In this section, we present the initial SEL domains and skills drawn from caregiver interviews, and the final Baka SEL framework after integrating items from the SEL literature and refining it with caregivers. We then explore the framework's components through an exploratory factor analysis of data from the caregiver SEL measurement tool, and comparison with other SEL frameworks.

#### The Baka SEL Framework

The initial domains and indicators drawn from the pile sort open coding of caregiver interviews during step 3 of phase 1 are summarized in Table 4 below. The positive and negative behavior indicators shown here were drawn directly from caregiver interview quotes. The domain titles and descriptions were written by the core research team to unite the behaviors into groups that resulted from the pile sort.

**Table 4:** Initial Baka SEL framework domains and indicators.

**Domain**	**Description**	**Positive indicators**	**Negative indicators**
Positive relationships with others	The child has good will toward others, is generous, and wants to do good things for others.	The child shares with others, especially food	The child does not share with others
		The child helps others to complete work	*No quote provided*
		The child is helpful/amenable toward others at home and outside the home.	*No quote provided*
		The child speaks kindly and politely to others	The child insults others
Respect for elders	The child is polite, respectful, and obedient toward elders	The child is polite toward elders	The child is not polite toward elders
		The child greets people who he/she sees	*No quote provided*
		The child does everything that elders ask of him/her	The child does not want to do what he/she is asked to do
		The child listens to the guidance of elders	The child does not listen to guidance of elders
		The child does errands/favors for elders	The child refuses to do errands/favors for elders
		The child does not address elders by their real names	*No quote provided*
Obedience	The child listens to others, does work at home, and is helpful/amenable toward others	The child does chores at home	The child just wants to play, and never wants to work
		The child listens to others	The child does not listen to what he/she is told
Conscien-tiousness	The child is aware of what he does, considers his impact on others, and thinks before making decisions	If provoked by others, the child does not react.	The child actively seeks out conflict and problems.
		The child inspires others to follow his/her example	The child reacts without thinking about his/her actions
		The child does quality work	The child causes a riot.
Peace	The child respects the bodies and property of others	The child does not steal	The child steals
		The child does not grab things from others	The child grabs things from others
		The child does not fight with others	The child fights with others

To complement indicators drawn from caregiver interviews, the team reviewed internationally validated SEL measures, to identify items that appear deeply important in Baka society, but which were mentioned by only a small number of caregivers. We identified six items:

1. The child is able to concentrate on tasks without getting distracted.
2. The child is able to calm him/herself down after becoming upset.
3. The child asks for forgiveness after hurting someone.
4. The child comforts a friend who is feeling sad or upset.
5. The child takes on new tasks without fear.
6. The child tries to do tasks independently without help, like washing and eating.

The core research team, particularly its three members who are Baka parents, felt these skills to be important in Baka culture, despite their infrequent or indirect appearance in caregiver interviews. The team opted to develop target behavior statements and non-mastery statements for each of these skills and include them in the first version of the questionnaire.

After follow-up interviews with caregivers to confirm the saliency of each domain and skill, we updated the framework to incorporate their feedback. The final Baka SEL framework in Table 5 below is the product of these progressive rounds of data collection, analysis, and reflection during phase 1, as well as refinements to SEL concepts that arose during phase 2 of the study.

**Table 5:** Baka SEL framework.

**Domain**	**Description**	**Target behaviors**
Positive relationships with others	The child has good will toward others, is generous, and wants to do good things for others.	• The child shares with others • The child speaks kindly to others • The child inspires others to follow his or her example
Respect for elders	The child is polite, respectful, and obedient toward elders	• The child is polite toward elders • The child greets elders who he or she sees • The child does errands and favors for elders • The child listens to the guidance of elders
Obedience	The child listens to others, does work at home, and is helpful/amenable toward others	• The child is helpful toward others at home and outside the home • The child does quality work
Conscien-tiousness	The child is aware of what he does, considers his impact on others, and thinks before making decisions	• The child does not react when provoked • The child does not fight with others • The child can calm him/herself when angry • The child asks for forgiveness after hurting someone
Peace	The child respects the bodies and property of others	• The child does not steal • The child helps others in need • The child understands others' feelings
Independence	The child seeks greater autonomy in daily activities.	• The child tries new things without fear • The child tries to bathe without parental help

#### Exploratory Factor Analysis

While exploratory factor analysis took place during psychometric validation of the caregiver SEL tool under phase 2, its results are presented here as they are important for understanding the Baka SEL framework. We found three underlying latent factors, each explaining approximately one-third of the variance in child scores. However, analysis output also indicated a good deal of variance in these items is unexplained by these three factors together. For this reason, we can only consider them as an exploratory thought exercise and not as definitive underlying constructs. Items with a correlation of >0.4 with the given factor are listed in Table 6 below. The factor titles are proposed by the research team to capture the essence of what may unite these skills into a single factor.

**Table 6:** Potential caregiver SEL factors.

**Factor 1: Emotional regulation**	**Factor 2: Responsibility**	**Factor 3: Conscience**
• The child does not fight with others • The child does not react when provoked • The child can calm him/herself when angry	• The child inspires others to follow his or her example • The child is polite toward elders • The child listens to the guidance of elders • The child does quality work • The child helps others in need	• The child shares with others • The child speaks kindly to others • The child does errands and favors for elders • The child does not steal

The grouping of skills by factor differs from the SEL framework resulting from the pile sort. The first factor, titled “Emotional regulation” points to children's ability to control their negative emotions and avoid violent conflict. The association between the items in the second factor is less obvious. We have opted for the title “Responsibility,” employing a two-pronged understanding of the word. Firstly, items appear to point to the child's ability to take responsibility for his or her actions, including to be a positive example to others, and to bring work tasks to completion. Secondly, items appear related to the child's tendency to take up societal responsibilities associated with children, including treating adults with politeness, listening to their guidance, and helping others in need. It is important to note that the term “politeness” here is an English approximation of the Baka word *titili*, which directly translates into “heaviness.” *Titili* can be understood similarly to the English idiom of not “treating someone lightly.” It encompasses a range of behaviors associated with respect for someone, including performing chores and errands for them, treating them with deference, and holding high regard for the advice they impart. The third factor seems to point to children's ability to uphold ethical values, such as sharing and obedience, and avoiding societal taboos such as insults and theft.

Viewing these factors through an ecological lens, they roughly correspond to the concentric environments that children experience. Emotional regulation pertains to the child's control over his or her own internal feelings and their immediate expression. Responsibility can be interpreted to relate to the child's direct interactions with children and adults, such as in the home or the school. Finally, Conscience appears related to the broader societal norms to which the child must learn to adhere. While these must be interpreted cautiously given remaining unexplained variance from the exploratory factor analysis, it is noteworthy that a child would logically develop skills within each factor at similar rates based on his or her progressive exposure to different social groups and environments.

### Building a Baka SEL Measurement Tool

In this section, we present the initial SEL tools, our findings from each round of piloting, and the final tools.

#### Initial Tools

##### Questionnaire Items

The research team developed initial teacher and caregiver Baka SEL measurement tools that included all domains and skills identified in the Baka SEL framework, with two exceptions. Questions about whether the child does chores and wants to bathe independently were included only on the caregiver questionnaire, as these relate exclusively to children's behavior in the home.

##### Administration Procedures

Ensuring tool validity required mitigating risk of caregiver bias, and attributing ratings based on real observed behavior. Assigning numerical ratings (e.g., on a Likert scale) to behaviors that caregivers are not accustomed to quantifying or discussing in absolute terms risks validity concerns. The team reflected on strategies from phase 1 that supported caregivers to be specific and open about child behavior, and referred to other existing SEL measures for inspiration. During phase 1, we found that caregivers relied on humor to diffuse tension when describing negative behaviors. Discussing behaviors in specific situations, rather than ascribing character judgments to children, helped caregivers to provide clear, detailed, and honest information. Review of existing SEL tools identified storytelling as a proven, comfortable, and accessible way for rural caregivers to talk about child behavior (37).

The research team therefore opted for a storytelling approach using two phases of questioning that prompt caregivers to describe their child's typical behavior in relevant contexts. First, the assessor asks an open-ended question about child behavior in a situation related to the overarching SEL domain, designed to elicit caregiver responses about the multiple target behaviors within that particular domain. Second, the assessor asks an open-ended question about each target behavior as needed to probe the caregiver to speak to a specific target behavior. The caregiver provides a verbal narrative response, to which the assessor assigns a quantitative evaluation. This approach promotes validity in three ways. Firstly, it allows caregivers to present information on their children's behavior by telling stories about them, which is familiar and natural. Second, it prompts them to think about specific situations, allowing caregivers to base ratings on observed behaviors. Third, it limits the number of interpretations at play of the meaning of the different levels of the rating scale to just those of the assessors, who have harmonized their understanding through the tool development process.

##### Scoring Protocols

The assessor coded the caregiver's response with a binary score of 1 or 0 based on whether the described behavior corresponds to the target behavior statement or the non-mastery statement. In terms of scoring procedure, the original tool included a binary scale, indicating whether the interviewee believes the child's behavior more closely corresponded to the target behavior statement or the non-mastery statement. For the caregiver questionnaire, the assessor rated the caregiver's response to the open-ended question. For the teacher questionnaire, the teacher was instructed to read the behavior statements and rate the child independently. The team chose this structure based on teachers having generally higher levels of literacy and greater familiarity with completing written forms than home caregivers. An example question is provided in Table 7.

**Table 7:** Example draft initial questionnaire item.

**How does your child react when he or she is unhappy?**	
7. Tell me about a time that another child wanted to fight with your child. How did he or she react?	□ If provoked by others, the child does not react. □ The child actively seeks out conflict and problems.
8. When your child is angry, is he or she able to calm down easily?	□ The child is able to calm him or herself down when he or she is angry. □ The child needs an adult to calm down when he or she is angry.

#### Updates to Tools From Initial Piloting

**Figure 2:**
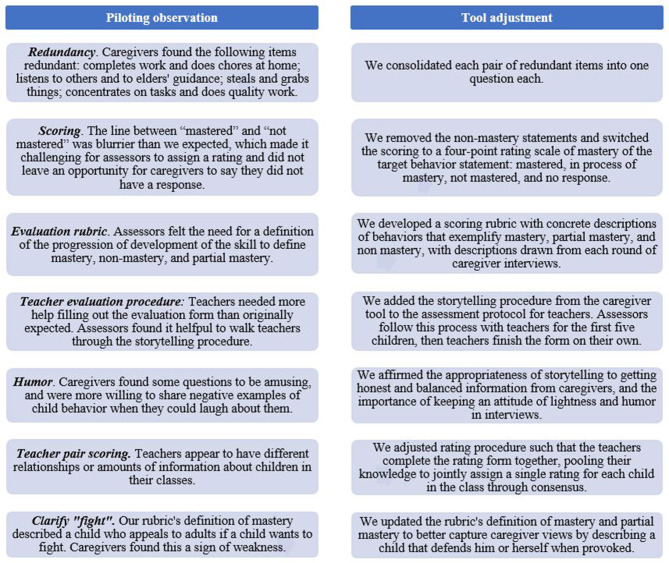
Observations and adaptations to tools after initial piloting.

The research team updated the tools to clarify the wording of questions, remove redundant items, and ensure that the scoring procedure is clear and feasible to administer based on initial piloting with five caregivers and two teachers. A summary of caregiver feedback and resulting tool updates are described in Figure 2.

The integration of an evaluation rubric supported common understanding of each mastery level, and consistency in scoring. Use of the rubric differs by tool. With the caregiver questionnaire, the assessor uses the rubric to assign a rating to the caregiver's response to the open-ended question. With the teacher questionnaire, the teachers use the rubric to assign ratings to child behavior. The assessor guides the teachers through this process for the first five children. The assessor first explains the evaluation rubric to teachers and gives them a copy. Similar to the caregiver questionnaire, the assessor asks the teachers the open-ended question, the teachers share a relevant story aloud, and the teachers review the rubric and assign the child a rating. The assessor serves as a sounding board for the teachers and offers explanations of the tool to help guide them to think of specific examples of the child's behavior, but does not influence their responses. After completing the first five children together, the teachers rate the rest of the class independently.

This exercise resulted in updated questionnaires for teachers and caregivers that endeavor to more faithfully capture child SEL development as Baka communities view it, ready for pilot testing with a larger sample for psychometric analysis. An example updated question from the caregiver and teacher questionnaire that shows the revised question structure with multiple mastery levels is shown in Table 8 below.

**Table 8:** Example revised questionnaire item.

**Questionnaire item**			
13. Helpfulness. Tell me about what your child does when someone needs help.		The child helps others in need. □ Mastered □ Partially mastered □ Not mastered □ No response	
**Question**	**Mastered**	**Partially mastered**	**Not mastered**
**Evaluation rubric**			
13. Tell me about what your child does when someone needs help.	The child reacts immediately when he/she notices that someone needs help. He/she helps others, often without being asked.	The child helps his or her friends first but does not always help others. He or she helps others if you ask, but not without being asked.	The child refuses to help others, even when asked. He or she prioritizes play and does not support others in need.

#### Full Piloting, Psychometric Analysis, and Tool Revisions

While we recognize the tools' inherent validity given that items were drawn directly from caregiver testimony, we sought to determine the tools' consistency and reliability as a measure of SEL through psychometric analysis. Table 9 summarizes quantitative findings for each tool, with interpretations in the sections below. Note that a score of 3 corresponds to mastery, 2 corresponds to in process of mastery, 1 corresponds to non-mastery, and 0 corresponds to no response.

**Table 9:** Quantitative findings of psychometric analysis of caregiver and teacher tool.

**Tool property**	**Teacher tool**	**Caregiver tool**
Children assessed	539	29
Teachers/caregivers interviewed	30 pairs	29
Score distribution	Normal	Normal
Mean	2.12	2.44
Standard deviation	0.33	0.20
Frequency of 3 rating	33%	49%
Frequency of 2 rating	42%	38%
Frequency of 1 rating	20%	10%
Frequency of 0 rating	5%	3%
Cronbach's alpha	0.73	Rater A: 0.6 Rater B: 0.68
Mean class alpha score:	0.48	N/A
Inter-rater reliability	N/A	0.91
Latent factors	1	3

##### Teacher Tool Piloting Results

Two core research team members administered the questionnaires to 30 teacher-pairs, with each teacher pair assigning a common rating to the child.

*Score distributions.* Teacher mean score of 2.12 and frequency of 2 rating point to a preference by teachers to score children in the middle of two extremes. This indicates the tool may not be capturing enough variance in children's progress from non-mastery to mastery.

*Interclass correlation.* Interclass correlation describes the amount of variance in scores accounted for by membership in a particular group—in this case, preschool class—as compared to variance attributable to differences between individuals in that group. We found large variability of student scores between and within classes, indicating systematic differences in teachers' interpretations of the questionnaire or views on SEL. There is need to clarify the tools' questions, rubric, and procedures to ensure consistent interpretation by teachers.

*Internal consistency.* Our Cronbach's alpha demonstrates that 73% of the observed variance in students' scores on the measure is accounted for by persistent variance of the underlying SEL constructs. This indicates that the measure has an acceptable level of internal consistency (39). Reviewing class-level alphas, we find a mean class alpha of 0.48, with half of classes above and the other half below the mean. While the measure has high internal consistency overall, some teacher pairs apply ratings with lower consistency than others. Consistency results further point to the need for more assessor norming and structured guidance to teachers to ensure consistent interpretations.

*Exploratory factor analysis.* Supposing the teacher-pairs with greater internal consistency are correctly applying the measure, we used their ratings to conduct an exploratory factor analysis to explore the dimensionality of the measure. The measure appears unidimensional, warranting the use of a single score for the measure. However, the factor analysis should be interpreted cautiously, as it is only based on data from teacher pairs with internal consistency at or above the mean.

**Figure 3:**
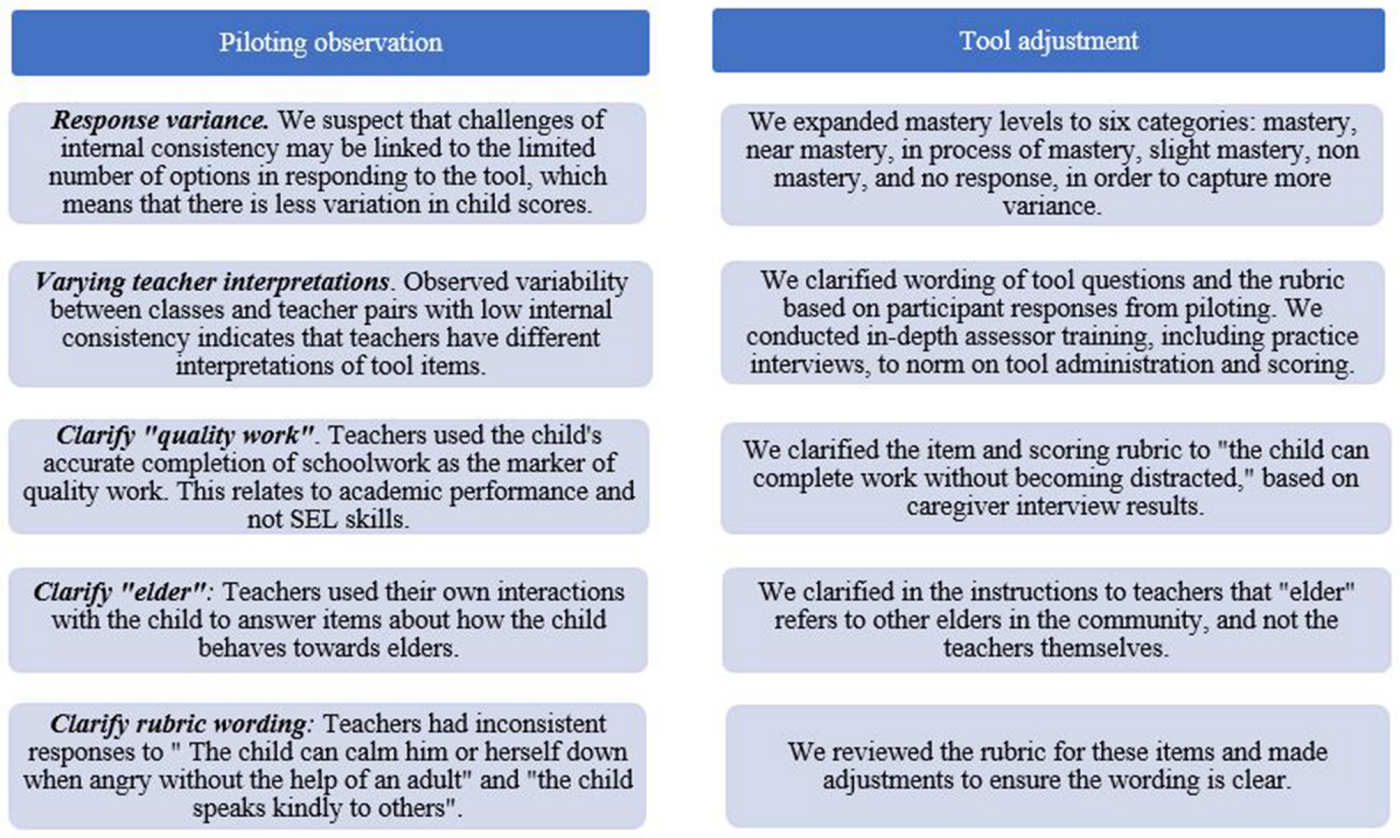
Updates to teacher tool post-piloting.

*Item test and item rest correlations.* We performed item-test and item-rest correlations to identify questions for which teachers assign ratings that do not correspond with the ratings they assign children on other questions. Assessors discussed these items to determine why this may be, and recommended updates for clarity and consistency summarized in Figure 3.

##### Adjustments to Teacher Tool

The design of the measure and the implementation of scoring adhere to recommendations drawn from the body of literature on SEL measurement, by having multiple raters collaborate on rating students to provide a more holistic measure of student's SEL. However, piloting revealed weaknesses on initial psychometric evaluations, pointing to the need to refine the tool to achieve uniform interpretation of questions and child behaviors. Based on piloting observations, we made changes to the teacher tool, summarized in Figure 3. The final tool is in Annex 1.

##### Caregiver Tool Piloting Results

Two members of the core research team (referred to as rater A and rater B) administered the tool to 29 caregivers, assessing 14 together for inter-rater reliability measurement, and 15 independently.

*Score distributions.* Caregiver mean score of 2.44, frequency of a 3 rating, and normal score distribution indicate that caregivers systematically rate children as having higher mastery levels than teachers.

*Internal consistency.* Each rater had acceptable consistency, with each rater's evaluations accounting for at least 60% of the variation in true underlying SEL skills. Caregiver ratings of child SEL skills consistently account for a higher amount of variance as compared with teacher ratings.

*Inter-rater reliability.* We found a 0.91 correlation between rater A and B's scores, indicating reliable interpretation and scoring of caregiver responses by the different raters.

*Exploratory factor analysis.* We found three underlying latent factors within the caregiver SEL measure, described in the section on the Baka SEL framework, which must be interpreted with caution.

**Figure 4:**
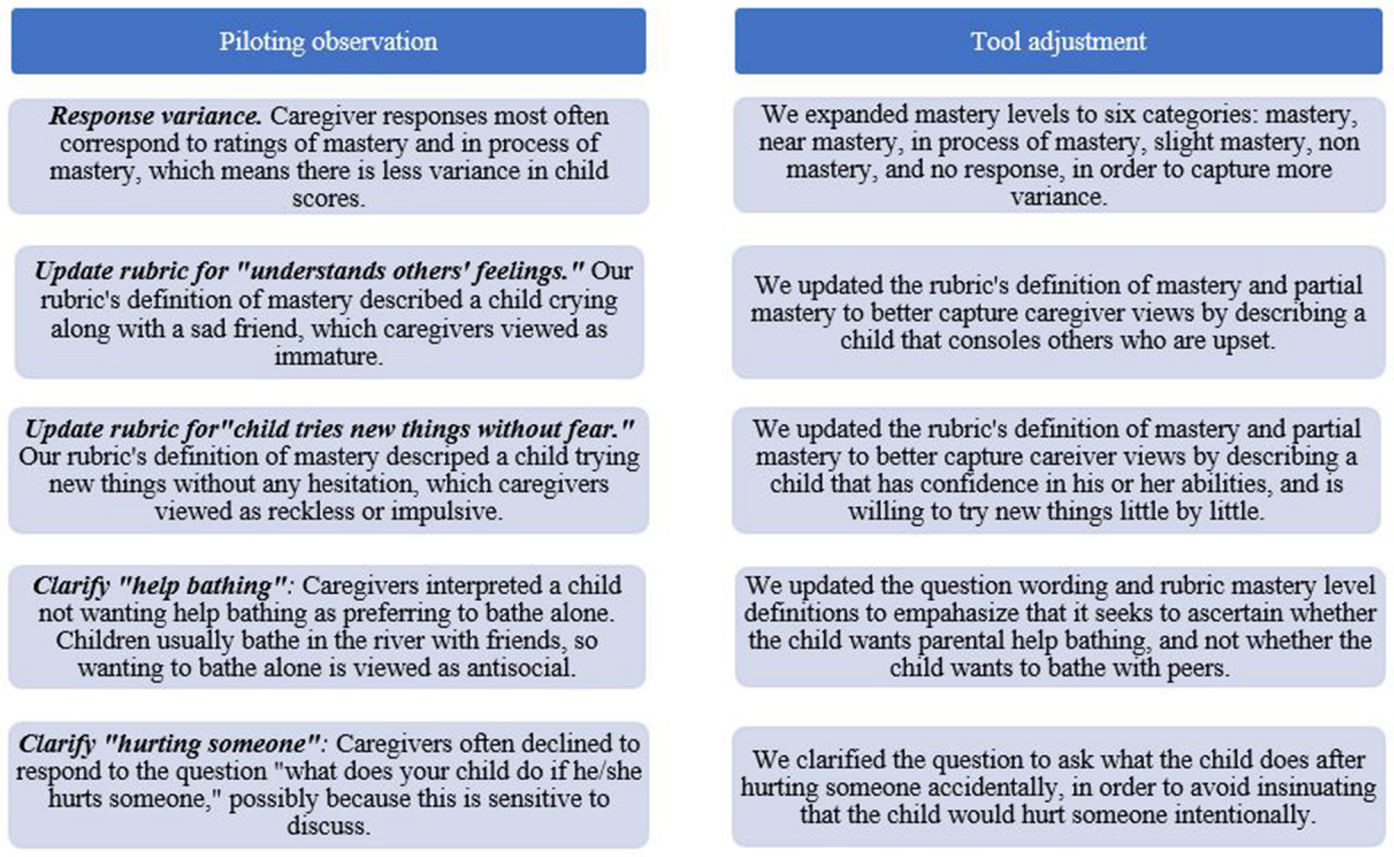
Updates to caregiver tool post-piloting.

*Item test and item rest correlations.* We reviewed item test and item rest correlations to identify questions for which caregiver interviews resulted in inconsistent ratings. Assessors reviewed these items, discussed their experience administering interviews, and recommended updates to questions and the scoring rubric summarized in Figure 4.

##### Adjustments to Caregiver Tool

Piloting revealed strong psychometric properties of the caregiver tool, and areas for improvement in clarity and consistency of interpretation. Based on piloting results, we identified updates to the caregiver tool as described in Figure 4. The updated tool is in Annex 2.

##### Comparison of Caregiver and Teacher Responses

To understand the relationship between teacher and caregiver scores, we compared their respective ratings for the same child. Data comparing teacher and caregiver scores for the same children is only available from the piloting phase of each tool, and as such does not reflect the adjustments targeting improved consistency that were incorporated after piloting.

**Figure 5:**
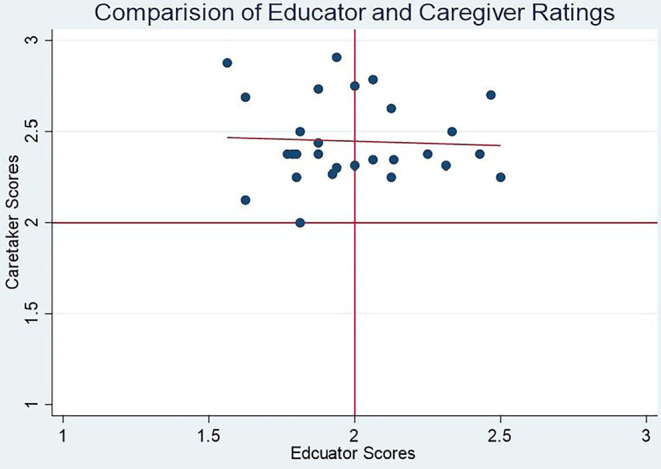
Comparison of caregiver and teacher scores for individual children.

A comparison of caregiver and teacher responses to their respective SEL questionnaires shows no correlation between their responses. Figure 5 shows scores for individual children rated both by teachers and caregivers, with teacher scores on the X axis and caregiver scores on the Y axis. As demonstrated by the upward skew of caregiver scores, teachers assigned children systematically lower scores than did caregivers, and have a higher spread in responses. The flat correlation line shows that receiving a higher score from a teacher has no relationship with receiving a higher score from a caregiver. Regressing caregiver ratings on teacher ratings shows no relationship between them.

Comparing caregiver and teacher responses to specific questions, we found that caregivers assign children higher ratings than teachers for approximately half of the test items, while teachers tend to assign children higher ratings than caregivers on just one question. Table 10 summarizes the questions for which caregivers rate children with higher, similar, or lower scores than teachers.

**Table 10:** Comparison of teacher and caregiver responses by item.

**Caregivers rated children higher than teachers**	**Caregivers and teachers assigned similarly varied ratings**	**Caregivers rated children lower than teachers**
The child speaks kindly to others The child does not fight with others The child is polite toward elders The child greets elders who he or she sees The child does quality work If people provoke him or her, the child does not react The child can calm him or herself down when angry without the help of an adult	The child shares with others The child inspires others to follow his or her example The child does errands/favors for elders The child listens to the guidance of elders The child asks for forgiveness after hurting someone The child does not steal The child helps others in need The child is compassionate and understands others' feelings	The child tries new things without fear

Even when caregivers and teachers assigned similarly varied scores in response to a given question, their responses still did not bear any relationship to one another. That is, a high caregiver rating on an item in the middle column did not correspond to a high teacher rating, and vice versa. While caregivers overall tended to assign children higher ratings than teachers there does not appear to be a clear trend in the types of questions for which caregivers tend to assign children higher ratings than teachers.

A revised description of SEL domains, open-ended questions, and target behaviors statements is in Table 11. The final retained evaluation rubric is in Annex 3.

**Table 11:** Revised questions and target behavior statements after initial piloting.

**Domain**	**Description**	**General question**	**Specific question**	**Target behavior statement**
Positive relationships with others	The child has good will toward others, is generous, and wants to do good things for others.	Tell me about how your child gets along with other children.	What happens when another child wants one of his things?	The child shares with others.
			How does your child talk to other children when they play?	The child speaks kindly to others.
			Tell me about what your child does when his or her friends are misbehaving.	The child inspires others to follow his or her example.
Respect for elders	The child is polite, respectful, and obedient toward elders.	How does your child behave toward elders?	Tell me about a time that your child spoke to elders	The child is polite toward elders.
				The child greets elders who he or she sees.
			What does your child do when an elder asks him or her to do an errand?	The child does all that elders ask of him/her.
			Tell me about a time when an elder gave your child advice.	The child listens to the guidance of elders.
Obedience	The child listens to others, does work at home, and is helpful and amenable toward others.	Tell me about when your child does work at home.	Tell me about how your child works at home.	The child is helpful toward others at home and outside the home.
			Does your child get distracted from his work?	The child completes work without getting distracted.
Conscien tiousness	The child is aware of what he does, considers his or her impact on others, and thinks before making decisions.	How does your child react when he or she is unhappy?	Tell me about a time when another child wanted to fight with him or her. How did he or she react?	If people provoke him or her, the child does not react.
			Tell me about a time when there was a disagreement with his or she her friends. How did he or she react?	The child does not fight with others.
			Tell me about a time when your child was angry. How did he or she calm down?	The child can calm him or herself down when angry without the help of an adult.
			Tell me about a time when he or she hurt someone by accident.	The child asks for forgiveness after accidentally hurting someone.
Peace	The child respects the bodies and property of others.	What happens when he or she wants something that does not belong to him or her?	Tell me about a time when he or she wanted something that belonged to someone else.	The child does not steal.
		How does your child perceive the needs of others?	Tell me about what your child she does when someone needs help	The child helps others in need.
			Tell me about a time that your child's friend was sad. What did he or she do?	The child is compassionate and understands others' feelings.
Independence	The child seeks to do things without help from an adult.	Does your child try to do things independently?	Tell me about a time that your child was faced with a new task that he or she had never tried. What did he or she do?	The child tries new things without fear.
			When your child bathes, does he or she want you to help?	The child tries to bathe without parental help.

## Discussion

The objective of this study was to test a participatory process for creating a culturally centered framework and measurement of SEL. We sought to answer two research questions: (1) How do Baka caregivers conceptualize SEL skills that children need for success? (2) How can a measurement tool faithfully and validly capture progress in developing these skills in early childhood?

Rather than propose an existing framework and adapt it to the Baka context, we built a framework from the ground up through multiple rounds of qualitative interviews with community members. In this section, we review our progress toward our original goals through these steps, the implications of our findings, and next steps to apply our findings in practice.

### Development of a Baka SEL Framework

Our first objective was to build a Baka SEL framework. We evaluate our success based on the framework's ability to capture caregiver perceptions of essential SEL skills, and to organize these into a logical framework.

The Baka SEL framework appears to faithfully summarize caregiver SEL priorities, owing to the participatory process of its development. We collaboratively developed the process for ascertaining caregiver perceptions of SEL by working with community members on the core and extended research team to refine our initial interview questions. Regarding the skills contained in the tool, we drew these from caregiver descriptions of desirable and undesirable behaviors and indicators of those behaviors. Multiple rounds of interviews about the SEL framework, and data collection using the measurement tools that are based on this framework, affirm that the framework reflects SEL skills that Baka caregivers view as priorities for their children to develop.

The accuracy of the Baka SEL tool's grouping of skills by domain is less clear. Our six proposed domains came from our pile sort open coding of caregiver statements on positive and negative child behaviors. Exploratory factor analysis of caregiver data points to three latent constructs, which appear to correspond with the child's mastery of social and emotional skills at the personal, interpersonal, and community levels. However, sizeable remaining unexplained variance means these latent factors should be interpreted with caution. The Baka SEL framework and exploratory factor analysis framework all share the foundational structure of emotional and relational skills that are salient throughout the literature, and seem to undergird SEL frameworks worldwide. Indeed, these are at the root of the Baka SEL framework's development, as they are embedded in our initial caregiver interview questions about children that have a “good head” and get along with others.

#### Comparisons With Other SEL Frameworks

We compared the Baka SEL framework with other global SEL frameworks to examine skills and behaviors that are distinct or shared between them. We used two predominant frameworks as points of comparison. The Collaborative for Academic, Social, and Emotional Learning (CASEL), formed in 1994, is a global leader in high-quality evidence based SEL (40, 41). The CASEL framework was developed and validated with American children, and its five SEL domains are summarized in Table 12. The Ecological Approaches to Social Emotional Learning (EASEL) Laboratory at the Harvard Graduate School of Education explores the relationship between SEL interventions and development in children, youth, families, and communities (42). EASEL's Taxonomy Project maps SEL skills across various frameworks in order to compare and analyze them, connect them to scientific evidence, and add clarity to global conversations on what constitutes SEL. Included frameworks represent a wide range of disciplines, are widely adopted, and include descriptions of skills, competencies, and attributes with sufficient detail to be coded. While frameworks are drawn from all over the world, there is a significant representation of frameworks from the United States and Europe. The Taxonomy Project has identified six SEL domains, summarized in Table 12. The rows in this table are not meant to suggest any linkage or comparability among the domains in the CASEL and EASEL frameworks.

**Table 12:** CASEL and EASEL frameworks.

**CASEL framework**		**EASEL framework**	
**Domain**	**Skills**	**Domain**	**Skills**
Self-awareness	Identifying emotions, accurate self-perception, recognizing strengths, self-confidence, and self-efficacy	Cognitive	Attention control, working memory and planning skills, inhibitory control, cognitive flexibility, and critical thinking
Self-management	Impulse control, stress management, self-discipline, self-motivation, goal-setting, and organizational skills	Emotion	Emotional knowledge and expression, emotional and behavioral regulation, and empathy/perspective taking
Social awareness	Perspective-taking, empathy, appreciating diversity, and respect for others	Social	Understanding social cues, conflict resolution and social problem solving, and prosocial and cooperative behavior
Relationship skills	Communication, social engagement, relationship-building, and teamwork	Values	Ethical values, performance values, civic values, and intellectual values
Responsible decision-making	Identifying problems, analyzing situations, solving problems, evaluating, reflecting, and ethical responsibility	Perspectives	Optimism, gratitude, openness, enthusiasm, and zest
		Identity	Self-knowledge, purpose, self-efficacy and growth mindset, and self-esteem

##### Commonalities Across Frameworks

The research undergirding the CASEL framework breaks SEL into emotional competence skills and relational or prosocial skills (43). We observe alignment between this breakdown and our initial interview questions that the extended community team helped us develop, which ask caregivers to describe a child with a “good head” who gets along well with others. “Good headedness” can be interpreted to refer to the child's emotional skills, while his or her ability to get along with others relates to relational skills. The latent factors revealed by exploratory factor analysis can be similarly categorized, with emotional regulation corresponding to emotional skills, and responsibility and conscience corresponding to relational skills. These threads also appear in EASEL, with the emotion, values, and identity domains classifiable as emotional competences, and the perspectives and social domains classifiable as social competencies. These commonalities appear to suggest the existence of salient latent concepts of what it means to be a respected and successful in a given society—emotional competence and interpersonal skills—and the possibility of universal SEL constructs that manifest themselves differently across cultures.

There are a number of specific SEL skill areas in common between the Baka, EASEL, and CASEL frameworks. While the Baka framework presents skills in specific and observable terms, the EASEL and CASEL frameworks list types of skill sets under each domain. Attention to others' feelings, and actions that elevate others' happiness and well-being, appear as common threads. Baka framework skills for positive relationships with others, including sharing, speaking kindly, and understanding others' feelings, are similar to social awareness skills under CASEL, and prosocial and cooperative behavior under EASEL. Furthermore, respect for others' bodies and property appear to be common expectations of children. Baka framework skills under the domain of peace, including not fighting or stealing, can be linked with ethical responsibility under CASEL and ethical and civic values under EASEL. This domain also corresponds to the conscience construct that emerged from the caregiver tool exploratory factor analysis, encompassing children's respect for societal norms and rules. Children's ability to manage their emotions, as well as their reactions to their emotions, are also shared across frameworks, reflected in commonalities between the CASEL self-management domain, the EASEL emotion domain, and the Baka conscientiousness domain. Management of emotions also appears in the emotional regulation construct from the caregiver tool exploratory factor analysis, whereby children ask for forgiveness, are able to calm down when angry, and refrain from retaliation in confrontations.

##### Differences Across Frameworks

The presence and absence of certain domains and skills in the Baka SEL framework distinguish it from CASEL and EASEL frameworks. Prevalent in the CASEL and EASEL frameworks are emphases on children's individual identities and self-awareness, including skills such as confidence, recognizing individual strengths, and having a sense of personal purpose. The Baka SEL framework includes independence, whereby children try new things and seek to do activities without adult assistance, but does not include attention to the child's own personality or individual goals. Featuring in the Baka SEL framework but not explicit in CASEL or EASEL frameworks are domains of obedience, respect for elders, independence, and responsibility.

Taken together, we observe that the Baka SEL framework displays high regard for skills and behaviors that support collaboration and community well-being, and notably lacks those related to individual identity and goals. Emphasis on collaboration and collective welfare aligns closely with the lack of hierarchies in Baka communities, and cultural value of shared prosperity over individualism. The Baka are a forager society and Baka culture emphasizes collective goals and well-being rather than focusing on the individual. Indeed, Dεngbε Bide teachers note that children engage most readily in collaborative games, and prefer working toward a common goal than competing to achieve individual results in class. Caregivers made clear in interviews that children are expected to contribute to household and community well-being. Child contributions include helping with housework, running errands, and being attentive to the needs of others. Children are expected not only to obey adult directives, but also to anticipate others' needs and proactively assist them. This is particularly true with regards to elders. While deference toward elders is absent from predominant global SEL domains, it features prominently in the Baka SEL framework. Caregivers noted that children should treat elders with *titili*, a term that evokes deference, politeness, attentiveness, and responsiveness to their needs and guidance. While contributing to collective welfare, Baka children are also expected to seek increasing independence from adults. Baka children spend significant parts of their days in the care and company of other children, learning and practicing new skills through lived experiences. Caregivers expect their children to readily attempt new activities and to manage their own care to the extent possible (such as bathing and eating independently).

Many global SEL frameworks, such the UNESCO Mahatma Gandhi Institute of Education for Peace, and the UNICEF Global Framework on Transferrable Skills, include skills in critical thinking, self-efficacy, identifying problems, and solving problems, which imply elements of challenging the status quo. These concepts are also missing from the Baka SEL framework (44, 45). The Baka SEL framework's consideration for obedience and respect for elders may result from the study's framing and perspectives. Our initial interview questions asked caregivers to describe a child who has a “good head” and gets along well with others, and a child who is a “head smacker” and does not get along well with others. While these questions yielded descriptions of child behavior that faithfully represent what caregivers believe children need to succeed, we recognize that data obtained in response center around adult perceptions of desirable child behavior. Resulting behaviors and domains clearly emphasize subservience and adherence to adult expectations. As all SEL frameworks are rooted in a cultural understanding of the world and children's role in it, we were unable in the scope of the present study to disentangle respondents' worldview and their views on skills children need for success.

Comparison of the Baka SEL framework with internationally validated SEL frameworks indicate commonalities across frameworks, as well as skills and domains unique to the Baka SEL tool. Overall, we observe that the Baka SEL tool prioritizes skills that promote collaboration, service, and deference to elders, and lacks skills that emphasize individual identity and purpose. Baka cultural norms emphasize collective well-being over individual achievement. The locus of Baka societal values and views on well-being lends critical importance to understanding SEL from the cultural perspective of the community at hand. Applying externally validated frameworks risks imposing norms and misrepresenting behaviors viewed as positive in one culture as being universally valuable.

### Meaningful Measurement of SEL Skills

Our second objective was to detect meaningful differences in SEL between children. For this, we reflect on the origins of our measured constructs, and psychometric analysis of our tool's performance during piloting.

With regards to our measured constructs, the Baka SEL measure captures the behaviors that caregivers believe are most important for children to be respected and successful in Baka society. It formulates them into questions to ascertain children's level of mastery of these target behaviors. This process was facilitated by diverse members of the Baka community, capturing a wide range of viewpoints and aspirations for children. Furthermore, the tool's structure reflects Baka cultural norms for talking about child behavior, using storytelling and humor to elicit honest reflections. This design ensures caregivers feel comfortable speaking about their child's development through concrete examples of behavior. The scoring rubric, also drawn from caregiver quotes on child behavior at different levels of mastery, supports consistency in quantitative coding of these qualitative responses. For this reason, we regard the measure as having high construct validity.

Psychometric data demonstrated that the teacher tool has moderate reliability and consistency, while the caregiver has high reliability and consistency. Teacher responses during piloting tended to cluster around the rating of “in process of mastery,” which limits the amount of variance that the tool can capture in children's growth. Teachers appear to have systematic differences in their ratings, with high variability in children's scores between classes as well as within classes. To support reliability and consistency of ratings in future tool administration, we expanded from a four-point scale to a six-point scale and clarified wording of the tool's questions, scoring procedures, and evaluation rubric. We will run psychometric analyses on subsequent rounds of data collection with the updated version in order to further strengthen the tool.

The caregiver tool demonstrates acceptable levels of internal consistency and high levels of inter-rater reliability. While normally distributed, caregiver ratings of children were systematically higher than those of teachers, indicating that caregivers view their children as having greater mastery of SEL skills than teachers. Differences between caregiver and teacher ratings may be attributable to differences in expectations for child behavior at home and at school, and differences in the relationships teachers and caregivers have with children. To improve the tool's reliability, we clarified wording of questions and evaluation rubrics for items with low or negative item test and item rest correlations. To capture more variance in responses, we expanded the tool from a four-point to a six-point scale as with the teacher tool.

We observed no correlation between teacher and caregiver ratings for the same child. While caregiver data are acceptably consistent and reliable, low consistency and reliability for teacher ratings mean that teacher scores should be interpreted with caution. The lack of relationship between teacher and caregiver scores indicates pervasive and systematic differences between how teachers and caregivers interpret the question items, and/or how they view children's mastery of SEL skills. All teachers interviewed are Baka themselves, and many are also parents, so it seems logical that they would have a similar understanding of desired SEL skills and of children's manifestations of target behaviors. Dεngbε Bide preschool centers intend to imitate the home environment so as to be welcoming to young learners.

However, research demonstrates that schools and households have different social norms. Despite its emphasis on community ownership, it is possible that Dεngbε Bide centers are no exception to this trend. Formal schooling in post-colonial societies such as Cameroon has historically retained distinct social and cultural environments from the societies they operate in. It is possible that views around schooling are strong enough that teachers, children, and/or caregivers associate even community learning spaces and local teachers with these expectations. Indeed, Dεngbε Bide centers serve in part to help children ease into a classroom-like environment before entering formal primary school. An aspect of this acclimation may be exposure to new norms for SEL. As the current tool was built from home caregivers' expectations around SEL, it is possible that it does not capture child mastery of target SEL behaviors in classroom settings. We intend for learners' classroom experiences to reinforce and reflect caregiver values. However, we recognize that teachers view child behavior in same-age groups in a semi-controlled classroom setting, whereas caregivers' experiences with children are in smaller and more flexible settings. Further qualitative interviews with teachers may yield supplemental items or evaluation rubric guidance that is specific to the Dengbe Bide classroom setting.

### Implications

We draw several key insights from our analysis with implications for how early childhood care and education programs should collaborate with communities to build SEL skills.

*SEL Skills Are Deeply Tied to Cultural Values.* Baka communities have SEL priorities that are distinct from dominant SEL frameworks. As Baka culture emphasizes respect for elders, responsibility to others and the community, empathy, and collective well-being, so does the Baka SEL framework. Predominant frameworks like CASEL that were standardized with American children highlight individual personality, goals, and successes, reflective of individualistic American society. Retrofitting an assessment based on the CASEL framework to the Baka risks categorizing children's lack of certain skills, such as recognizing personal strengths or setting personal goals, as developmental deficits rather than assets.

*There Are Common Underlying Constructs Across SEL Frameworks.* The Baka SEL framework shares skills, domains, and fundamental constructs with other predominant SEL frameworks, such as understanding and regulating one's own emotions, attentiveness and responsiveness to others' feelings, respect for others' bodies and property, and cooperation with others. SEL skills and domains can be analyzed across multiple axes. Literature on SEL categorizes them into emotional or relational skills. Our exploratory factor analysis groups them by ecological level: from self-management to interpersonal relationships to respect for societal values. While the specific behaviors that children exhibit in each cultural context may differ, these components appear present across SEL frameworks, and indicate shared foundational elements of social and emotional competence across societies.

*Even in Community Preschool Centers, Systematic Differences Exist Between Home and School SEL.* Teacher and caregiver ratings of children in response to the Baka SEL measurement tools were uncorrelated. Despite growing up and living in the same cultural and environment, teachers and caregivers applied different ratings to children. This may be attributed to different expectations for child behavior in school and at home, children acting differently in each space, or teachers and caregivers having different types of relationships with children. Measuring SEL skills in just the school environment may omit children's strengths in the home environment, and the skills that caregivers wish them to develop.

*Child Scores on Nascent SEL Measures Should Be Interpreted and Applied With Caution.* While the tools have high construct validity, the teacher tool has limited reliability. Teachers and caregivers rated the same child differently on tool items. Further rounds of data collection will be necessary to strengthen tool performance and gather enough data to set parameters for normal child behavior. Only after this is done can we attempt to use these tools to measure child SEL development over time and identify children at-risk or underperforming,

*Storytelling Is an Effective Assessment Approach.* Caregivers and teachers readily used storytelling to share concrete examples of a broad spectrum of positive and negative child behavior. This approach allows caregivers to describe a child's specific actions rather than pass general judgments of the child's skills or character. Humor in sharing stories put caregivers at ease to describe negative behaviors. It also enables them to provide narrative information about the child, rather than assigning a numerical rating to complex and subjective concepts. Assessors were able to use clear rubrics and norming sessions to arrive at consistent tool interpretation, and assign quantitative scores to teacher and caregiver qualitative responses to generate acceptably consistent and reliable ratings of child SEL.

*Localizing SEL Is Feasible and Worthwhile.* While development of the SEL measure took time, developing the SEL framework was quick and simple. Development of the Baka SEL framework took place over a period of 2 weeks, from initial data collection through verification of the framework's items. Development of the measurement tool extended over 4 months. With essential elements of the questionnaire structure, like the use of storytelling, scoring by mastery levels, and a rubric to concretely describe these levels, development of subsequent tools will likely be more efficient. We strongly encourage early childhood care and education programs short on time to, at minimum, collaborate with communities to build a localized framework for SEL. Understanding community SEL priorities is essential for ensuring that the program does not impose cultural values. It is particularly critical for programs working with marginalized and indigenous populations to avoid colonizing SEL through their programming.

### Next Steps

In the coming months, we will move toward accomplishing the other two goals of our study: integrating skills from the Baka SEL framework into the Dεngbε Bide curriculum, and measuring changes in children's SEL skills over time.

#### Integration of SEL Into the Curriculum

We recognize that SEL development takes place largely in the home. However, we also recognize that schools can reinforce systemic racism and discrimination, and that schools have advanced cultural erasure through targeted assimilation of indigenous children into the mainstream. Respecting indigenous identities requires actively pushing back on these forces by emphasizing the language, values, and culture of Baka communities. For this reason, we will integrate SEL skills from the Baka SEL framework explicitly and implicitly into the curriculum. Our target entry points include reviewing the SEL framework with teachers during their annual trainings, developing children's stories that center around SEL messaging, and developing educational songs that present scenarios and refrains that help children learn and remember SEL skills. These steps will entail participatory dialogue by our core research team with Dεngbε Bide teachers and our extended community team.

#### Measuring Changes in SEL Skills Over Time

Establishing reliable scoring is an important prerequisite to measuring children's progress over time. While the caregiver tool performed adequately in piloting, the teacher tool's psychometric properties demonstrate that it requires strengthening before it can be considered to faithfully capture children's mastery of SEL skills. We have adjusted the tool to improve its consistency and reliability, and recognize teacher ratings may only be applicable to children's SEL skills in the classroom environment. After testing improvements and stabilizing both tools, we will use data from subsequent assessment exercises to determine the degree to which the tool is sensitive to changes in SEL skills over time. We will administer each tool at different intervals. Applying the teacher tool at the beginning and end of each school year will allow Dεngbε Bide to have a snapshot of SEL skills for all participating children at baseline and endline, providing two data points to track growth. We will interview a small sample of caregivers with the caregiver tool during monthly program monitoring visits. Over the course of the year, we will endeavor to assess all participating children through the caregiver tool at least once. This data will allow Dεngbε Bide to have a continuum of information on progressions in children's SEL skills throughout the year, and qualitative information on caregivers' views on child SEL development. We will integrate examples from caregiver and teacher storytelling into the evaluation rubric, in order to provide more concrete examples and support continuous improvement in scoring reliability.

### Limitations

Study limitations include translation across multiple languages, lack of reliable data on child age, and limited ability to gather follow-up data using the revised SEL tools.

Much of the desk research that informed initial core team discussions around SEL was available only in English, while a good deal of the French research was based on SEL frameworks originally developed in English. The English-speaking core team member translated and explained English language research to the rest of the core team, and meaning may have been distorted or lost along the way.

As SEL skills tend to increase with age, correlation between age and score is an indicator of a SEL measure's convergent validity. Almost all children in the target population were born in the home and not in a hospital, and so they lack records of an exact date of birth. Caregivers tend to estimate age based on visible characteristics, so child ages per parent report reflect developmental traits. Furthermore, these estimates are expressed in years and not months, so there is very little variability upon which we could regress SEL scores. For this reason, the relationship between SEL scores and age remains a gap in our analysis.

Finally, it was intended that the research team would implement updated versions of the teacher and caregiver tool during the final trimester of the 2019–2020 school year in Dεngbε Bide preschool centers. However, school closures due to COVID-19 limited our ability to gather data. The present paper discusses only the findings of the pilot, and does not include psychometric information about the final revised tools.

## Data Availability Statement

The raw data supporting the conclusions of this article will be made available by the authors, without undue reservation.

## Ethics Statement

Ethical review and approval was not required for the study on human participants in accordance with the local legislation and institutional requirements. The patients/participants provided their written informed consent to participate in this study.

## Author Contributions

BA and SS led study design, data collection, qualitative data analysis to construct the SEL framework, conversion of the framework into an assessment tool, and piloting of assessment tools. SS conducted the literature review, coordinated qualitative and quantitative analysis, coordinated among study team members, and translated findings into English. AS provided overall guidance to study design and data analysis. PC conducted psychometric analysis of tool piloting data and generated recommendations for tool strengthening. All authors contributed to the article and approved the submitted version.

## Conflict of Interest

The authors declare that the research was conducted in the absence of any commercial or financial relationships that could be construed as a potential conflict of interest.

## Supplementary Material

Annex 1 Teacher Questionnaire.

Annex 2 Caregiver Questionnaire.

Annex 3 Evaluation Rubric.
