# Supplementary material for: Without Assumptions: Development of a Socio-Emotional Learning Framework That Reflects Community Values in Cameroon
Source: Front Public Health. 2021 May 7;9:602546. doi: 10.3389/fpubh.2021.602546 (PMC8137823; doi:10.3389/fpubh.2021.602546)
Supplement: Annex 1 — Teacher Questionnaire. [file Table_1.pdf]

Village__________ Niveau_________ Date_______ Animateur 1_____________ Animateur 2_____________

Questionnaire à l’Animateur – Développement Socio-Emotionnel de l’Enfant

1. Listez tous les enfants dans votre classe dans le tableau « REPONSES ».
2. Référez-vous aux Niveaux de Maitrise. Lisez la Question de Réflexion. Racontez à votre collegue une histoire spécifique qui sert d’un exemple des habitudes normales de l’enfant dans la situation donnée.
3. Décidez ensemble si l’histoire montre que le comportement est maitrisé (5), presque maitrisé (4), en cours de maitrise (3), un peu maitrisé (2), non-maitrisé (1), ou si vous ne savez pas (0). Notez l’enfant dans le tableau.

#	QUESTIONS
1	Yandɛ é à gapà èé kɛ̀ e tɛ nɛ̀ tɛ lɛ biɛ̀ Yandɛ o. *L’enfant partage avec les autres enfants.*
2	Yandɛ bà ngomà tɛ lɛ̀ biɛ Yandɛ o jókò (E jókò bo). *L’enfant parle bien aux autres enfants (est gentil).*
3	Yandɛ éà gbiɛ lɛ̀ biɛ̀o à na tomà jokò na dotò a ngɛó. *L’enfant amène les autres à suivre son exemple.*
4	Yandɛ é à tɔ tìtili pe kobo na gbao. *L’enfant est poli envers des grandes personnes de la communauté.*
5	Yandɛ é à jukò koboo kobo na gbao. *L’enfant salut grandes personnes de la communauté.*
6	Yandɛ é à mɛɛ̀ èe kɔ̀pɛ kɛ̀ kobo na gbao wó à manà pɛ́ nɛ̀. *L’enfant fait tous que les grandes personnes du village lui dit.*
7	Yandɛ bà jɛ lewù kɛ̀o kobo na gbao wó à manà pɛ́ nɛ̀. *L’enfant écoute les conseils des grandes personnes du village.*
8	Yandɛ bà mɛɛ̀ bèlà a ngɛ́ jókò (bèlà kɛ̀ é mɛɛ̀ jókò nɛ̀). *L’enfant fait bien son travail (un travail de qualité).*
9	Pe boo wá dɔ tɛ we, Yandɛ ndé na gelo. *Si les gens viennent avec les problèmes, l’enfant ne réagit pas.*
10	Yandɛ é mɛɛ́ kɔlɛ tɛ biɛ̀ lɛ̀ Yandɛo ode. L’enfant ne bagarre pas avec les autres enfants.
11	Yandɛ tɛ na njiì a kɛ na jɛ̀ kabu ndé pe kobo à yangɛ́. *L’enfant peut se calmer après qu’il/elle se fâche, sans l’aide d’un adulte.*
12	Éà gbɔ bibi tɛ bo à tie kɛ̀ a mɛɛ̀lɛ̀ sítí tɛ bo nɛ̀. *L’enfant demande pardonne après avoir fait du mal à quelqu’un.*
13	Yandɛ é gboò éeo ode. *L’enfant n’arrache pas des choses.*
14	Yandɛ bà yangà boo a bèlà a ngó. *L’enfant aide les autres en besoin.*
15	Yandɛ é tɛ bumɛ na yuwà, éà jɛ̀ pe na biyɛ́kɛ̀ bíɛ̀ bo bà jɛ̀ nɛ̀. *L’enfant a un cœur de pitié. Il comprend comment les autres se ressent.*
16	Yandɛ bà lɛ́ bèlà ná tɔtɔo ndé na jɛ̀ gò. *L’enfant tente des nouvelles activités sans peur.*

REPONSES

#	Nom de l’enfant	5 = Maitrisé ; 4 = Presque maitrisé ; 3 = En cours de maitrise ; 2 = Un peu maitrisé, ; 1 = non-maitrisé ; 0 = ne sait pas.															
		1	2	3	4	5	6	7	8	9	10	11	12	13	14	15	16
1		´															
2																	
3																	
4																	
5																	
6																	
7																	
8																	
9																	
10																	
11																	
12																	
13																	
14																	
15																	
16																	
17																	
18																	
19																	
20																	
21																	
22																	
23																	
24																	
25																	
26																	
27																	
28																	
29																	
