# Supplementary material for: Without Assumptions: Development of a Socio-Emotional Learning Framework That Reflects Community Values in Cameroon
Source: Front Public Health. 2021 May 7;9:602546. doi: 10.3389/fpubh.2021.602546 (PMC8137823; doi:10.3389/fpubh.2021.602546)
Supplement: Annex 2 — Caregiver Questionnaire. [file Table_2.pdf]

Questionnaire des Parents – Développement Socio-Emotionnel de l'Enfant

Village _________________ Date ___________

Nom de l'enfant _______________ Age de l'enfant_____________ Sexe de l'enfant________

**Kɛ là mò é à doto tɛ a mɛɛ̀ sɔ̀lɔ á to biɛ̀ yandɛo pe?**<br>***Parle-moi de comment il joue et s'entend avec les enfants.***	
1. Kè e bà wɔ́tɔ̀ pe a tie kɛ̀ nɔɔ̀ biɔɔ wó à jɛ̀ te gomò èe a ngɛ́ nɛ̀ ?<br>***Partager.*** *Qu'est-ce qui se passe quand un autre enfant veut de ses choses ?*	É à gapà eè kɛ̀ e tɛ nɛ tɛ lɛ̀ biɔɔ.<br>*L'enfant partage avec les autres enfants*<br>□ Maitrisé □ Presque maitrisé □ En cours de maitrise<br>□ Un peu maitrisé □ non-maitrisé □ ne sait pas.
**2.** Yandɛ é à ngomà tɛ biɔɔ pe ?<br>***Insulter.*** *Comment est-ce que ton enfant parle aux amies quand il joue ?*	É à ngòmà tɛ biɔɔ jókò.<br>*L'enfant parle bien aux autres enfants (est gentil).*<br>□ Maitrisé □ Presque maitrisé □ En cours de maitrise<br>□ Un peu maitrisé □ non-maitrisé □ ne sait pas.
3. Tɛ pe làa mò bà lɛ lòtì a ngɛ́ó ? Bà pe wá mɛɛ̀ èe kɛ̀ e jókò a mɛɛ̀ de nɛ̀ ?<br>**Imiter** : Est-ce que ton enfant imite souvent ses amies ? Même quand ils font quelque chose qu'il ne faut pas faire ?	É à gbiɛ̀ lɛ̀ biɔ à na tomà jókò na dotò a ngɛ́ó.<br>*L'enfant amène les autres à suivre son exemple.*<br>□ Maitrisé □ Presque maitrisé □ En cours de maitrise<br>□ Un peu maitrisé □ non-maitrisé □ ne sait pas.
**Kɛ̀ là a mò e a dòto a la koboo pe ?**<br>***Comment est-ce que ton enfant se comporte envers des grandes personnes ?***	
**4 et 5.** Kà là a mò bà ngomà á là kobo à boo pe ?<br>***Parler***. *Comment est-ce que ton enfant parle aux grandes personnes ?*	**4.** É à tɔ tìtili pe koboo.<br>*L'enfant est poli envers des grandes personnes*<br>□ Maitrisé □ Presque maitrisé □ En cours de maitrise<br>□ Un peu maitrisé □ non-maitrisé □ ne sait pas.<br>**5.** É à jukò koboo a kpàje à tie kɛ̀ wó tɔ wó à makà tɛ nɛ̀.<br>*L'enfant salut les grandes personnes gens quand il les voit.*<br>□ Maitrisé □ Presque maitrisé □ En cours de maitrise<br>□ Un peu maitrisé □ non-maitrisé □ ne sait pas.
**6.** Là a mò e a mɛɛ̀ pe à tie kɛ koboo wo a kindɛ́ tɛ a tima nɛ̀ ?<br>***Commissions***. *Quand une grande personne lui demande de faire une commission, qu'est-ce qu'il fait ?*	É à mɛɛ̀ eè kɔ̀pɛ kɛ̀ kobò wó à manà pe nɛ̀.<br>*L'enfant fait tous que les grandes personnes lui dit.*<br>□ Maitrisé □ Presque maitrisé □ En cours de maitrise<br>□ Un peu maitrisé □ non-maitrisé □ ne sait pas.
**7.** Ke là a mò bà mɛɛ̀ pe a tie kɛ koboo wo à tɔ pɛ lewu nɛ̀ ?<br>***Ecouter.*** *Quand une grande personne le donne des conseils, qu'est-ce que ton enfant fait ?*	E yandɛ kɛ é a jɛ̀ lewu kɔpɛ kɛò koboo à manà pɛ́ nɛ̀.<br>*L'enfant écoute les conseils des grandes personnes.*<br>□ Maitrisé □ Presque maitrisé □ En cours de maitrise<br>□ Un peu maitrisé □ non-maitrisé □ ne sait pas.
**Kè là a mò bà mɛɛ̀ bèlà nà ndao pe ?**<br>***Comment est-ce que ton enfant travail à la maison ? Parle-moi de comment ton enfant travail à la maison***	
**8.** Tɛ pe là a mò bà mɛɛ̀ bèlà a nda tie kɔ̀pɛ̀ ?<br>***Travailler***. *Est-ce que ton enfant fait des travaux régulièrement à la maison ?*	É à yangà boo a ngɛ́ó a bèlà nà ndao tɛ nɔɔ̀ lɛ̀ bèlào<br>*Il aide les gens dans les travaux de la maison et bien d'autres.*<br>□ Maitrisé □ Presque maitrisé □ En cours de maitrise<br>□ Un peu maitrisé □ non-maitrisé □ ne sait pas.
18. A tie kɛ̀ làa mò bà mɛɛ̀ tɛ bèlà nɛ̀, moà sià pe e mɛɛ̀ jókò à tie kɔ̀pɛ ?<br>***Qualité.*** *Est-ce que ton enfant finit ses travaux sans se distraire ?*	Yandɛ bà nɔɔ̀ jókò à tie kɛ̀ é à mɛɛ̀ tɛ bèlà.<br>*L'enfant fait bien son travail sans distraction.*<br>□ Maitrisé □ Presque maitrisé □ En cours de maitrise<br>□ Un peu maitrisé □ non-maitrisé □ ne sait pas.
**Ke là a mò bà mɛɛ̀ pe a tie kɛ é a jɛ̀ tɛ kabu nɛ ?**<br>***Comment est-ce que ton enfant réagit quand il n'est pas content ?***	
**9.** Ke e mɛɛ̀ ngi pe a tie kɛ̀ nɔɔ̀ yandɛ̀ ɔɔngi à gelɛ tɛ kɔlɛ nɛ̀ ?	**9.** Biɔ wó ko na gelɛ tɛ we é à dòto dòto ndé na gelo.<br>*Si les gens viennent avec les problèmes, l'enfant ne réagit pas.*<br>□ Maitrisé □ Presque maitrisé □ En cours de maitrise

Agir. *Parle-moi d'un temps qu'un autre enfant voulait bagarrer avec lui. Comment a-t-il réagi ?*	□ Un peu maitrisé □ non-maitrisé □ ne sait pas.<br>**10.** É geè lɛ̀ biɔɔ tɛ kɔlɛ ode.<br>*L'enfant ne bagarre pas avec les autres enfants.*<br>□ Maitrisé □ Presque maitrisé □ En cours de maitrise<br>□ Un peu maitrisé □ non-maitrisé □ ne sait pas.
**11.** A tie kɛ̀ yandɛ bà jɛ̀ tɛ kabu nɛ̀, é à mɛɛ̀ pe à na bɛ tɛ kabu.<br>***Calmer***. *Quand ton enfant se fâche, est-ce qu'il/elle se calme facilement ?*	Yandɛ e tɛ tie na njiì a kɛ na jɛ̀ kabu ndé pe kobo a yangɛ́.<br>*L'enfant peut se calmer après qu'il/elle se fâche, sans l'aide d'un adulte.*<br>□ Maitrisé □ Presque maitrisé □ En cours de maitrise<br>□ Un peu maitrisé □ non-maitrisé □ ne sait pas.
**12.** E à mɛɛ̀ pe à tie kɛ̀ a mɛɛ̀ lɛ tɛ èe nà sítí tɛ bo nɛ̀ ?<br>**Pardonner**. Que fait-il quand il fait du mal à quelqu'un ?	É à gbɔ bibi tɛ bo a tie kɛ̀ é à mɛɛ̀ tɛ siti nɛ̀.<br>*Il demande pardonne après avoir fait du mal à quelqu'un*<br>□ Maitrisé □ Presque maitrisé □ En cours de maitrise<br>□ Un peu maitrisé □ non-maitrisé □ ne sait pas.
**Là a mò bà mɛɛ̀ pé à tie kɛ̀ e à ye tɛ eè a boo nɛ̀ ?**<br>***Comment est-ce que ton enfant fait quand il/elle veut quelque chose qui ne l'appartient pas ?***	
**13.** Manà pe èe kɛ̀ é à mɛɛ̀ nɛ̀ a tie kɛ̀ é à ye tɛ èe a boo nɛ̀.<br>***Arracher***. *Parle-moi de ce qu'il/elle fait quand il/elle veut quelque chose qui appartient à d'autrui.*	É gboò eè a biɔ ndé na yì ode<br>*L'enfant n'arrache pas des choses*<br>□ Maitrisé □ Presque maitrisé □ En cours de maitrise<br>□ Un peu maitrisé □ non-maitrisé □ ne sait pas.
**Kè là a mò e bà mɛɛ̀ pe à na sià gomò è a biɔ ?**<br>***Comment est-ce que ton enfant perçoit les besoins des autres ?***	
**14.** Manà pè èe kɛ e a mɛɛ̀ à tie kɛ̀ nɔɔ̀ bo a ye yangà nɛ̀ ?<br>**Aider.** Parle-moi de ce qu'il fait quand quelqu'un a besoin d'aide.	Yandɛ ná é bà yangà boo a bèlà a ngó.<br>*L'enfant aide les autres en besoin.*<br>□ Maitrisé □ Presque maitrisé □ En cours de maitrise<br>□ Un peu maitrisé □ non-maitrisé □ ne sait pas.
**15.** Kè là a mò e bà mɛɛ̀ pe à tie kɛ̀ biɛ̀ bo tɛ a yuwa nɛ̀ ?<br>**Compatir.** Qu'est-ce que ton enfant fait si son ami est triste ?	Yandɛ na bumɛ na yuwa.<br>*L'enfant a un cœur de pitié. Il comprend comment les autres se ressent.*<br>□ Maitrisé □ Presque maitrisé □ En cours de maitrise<br>□ Un peu maitrisé □ non-maitrisé □ ne sait pas.
**Te pe là a mo, e a ye pe e mɛɛ̀ èe a ngɛ ngɛ mòmolo nde a ye pe wo yangɛ̀ ?**<br>**Est-ce que ton veut faire des choses indépendamment ?**	
16. É a mɛɛ̀ pe à tie na mɛɛ̀ nɔɔ̀ èe kɛ̀ é mɛlɛ so nɛ?<br>***Tenter*** *: Comment agit-il face à une nouvelle tache ou activité qu'il n'a jamais fait ?*	Yandɛ na e tɛ kpɛ́kɛ́ na ye na mɛɛ̀ èe na tɔ̀tɔ.<br>*L'enfant tente des nouvelles activités sans peur.*<br>□ Maitrisé □ Presque maitrisé □ En cours de maitrise<br>□ Un peu maitrisé □ non-maitrisé □ ne sait pas.
17. A tie kɛ̀ é à nyɛ̀ tɛ ngo nɛ̀, tɛ é à ye pe nɔɔ̀ boo e yangɛ́ ?<br>***Se laver.*** *Quand il/elle se lave, est-ce qu'il/elle veut que tu l'aide ?*	Yàndɛ bà ye na nyɛ̀ ngo nde na ye yanga koboo (a bo nɔɔ̀ tie e tɛ a ye pe wó yangɛ́).<br>*L'enfant veut se laver sans l'aide de ses parents (mais peut parfois avoir besoin d'appui).*<br>□ Maitrisé □ Presque maitrisé □ En cours de maitrise<br>□ Un peu maitrisé □ non-maitrisé □ ne sait pas.
