# Supplementary material for: Without Assumptions: Development of a Socio-Emotional Learning Framework That Reflects Community Values in Cameroon
Source: Front Public Health. 2021 May 7;9:602546. doi: 10.3389/fpubh.2021.602546 (PMC8137823; doi:10.3389/fpubh.2021.602546)
Supplement: Annex 3 — Evaluation Rubric. [file Table_3.pdf]

Niveaux de Maitrise en Développement Socio-Emotionnel

Question de réflexion	Comportement cible/attendu	Maitrisé (Le comportement est bien, n'est pas problématique)	En cours de maitrisé (Parfois bien, mais un peu problématique)	Non maitrisé (Le comportement est souvent problématique)
**1. Kè nye bà wɔtɔ̀ à tie kɛɛ nɔɔ̀ yandɛ éà jɛ̀ tɛ gomò èe a ngɛ́ nɛ̀ ? Partager.** *Qu'est-ce qui se passe quand un autre enfant veut de ses choses ?*	**Yandɛ é à gapà èé kɛ̀ e tɛ nɛ̀ tɛ lɛ biɛ̀ Yandɛ o.** *L'enfant partage avec les autres enfants.*	L'enfant partage librement ses choses avec les autres.	L'enfant partage parfois, mais il y a des fois et/ou des choses qu'il ne veut pas partager	L'enfant ne partage pas, ne veut pas que les autres touchent ses choses.
**2. kè é à ngomà tɛ lɛ̀ biɔ pe à tie kɛ̀ wó tɔ wó à mɛɛ̀ tɛ sɔ̀lɔ nɛ̀ ? Insulter.** *Comment est-ce qu'il parle aux amies quand il joue ?*	**Yandɛ bà ngomà tɛ lɛ̀ biɛ Yandɛ o jókò. (E jókò bo)** *L'enfant parle bien aux autres enfants (est gentil)*	L'enfant joue tranquillement avec les autres, il joue sans insulter et sans se fâcher.	L'enfant a parfois des difficultés à s'entendre avec les autres, se fâche contre ses amies.	L'enfant ne joue pas bien avec les autres, insulte les autres enfants.
**3. Tɛ pe á yo na lɛ lɛ̀ biɛ̀o bà pe wó à mɛɛ̀ nɔɔ̀ èe kɛ̀ e jókò a mɛɛ̀ de nɛ̀ ? Imiter :** *Est-ce qu'il imite souvent ses amies, même quand ils font quelque chose qu'il ne faut pas faire ?*	**Yandɛ éà gbiɛ lɛ̀ biɛ̀o à na tomà jokò na dotò a ngɛó.** *L'enfant amène les autres à suivre son exemple.*	L'enfant agit selon sa façon de voir les choses, il agit bien même quand les autres n'agissent pas bien.	L'enfant peut parfois faire des mauvaises choses, mais parfois il se rende compte de ses actions et se corrige.	L'enfant imite les autres qui font des mauvaises choses
**4 et 5. Kè éà ngomà tɛ kobo nà boo pe ? Parler.** *Comment est-ce qu'il parle aux grandes personnes ?*	**4. Yandɛ é à tɔ tìtili pe** kobo na gbao**.** *L'enfant est poli envers des grandes personnes de la communauté.*	L'enfant parle toujours bien aux grandes personnes avec du respect et avec politesse. L'enfant n'insulte jamais des grandes personnes de la communauté.	L'enfant est un peu poli aux grandes personnes du village, mais pas toujours. Il les insultait avant, mais commence à ne plus le faire. Il peut le faire de temps en temps mais il se corrige.	L'enfant parle mal de ses parents et insulte les grandes personnes de la communauté, sans se corriger.
	**5. Yandɛ é à jukò** kobo na gbao **à tie kɛ̀ wó tɔ́ wó à makà tɛ nɛ̀.** *L'enfant salut les grandes personnes du village quand il les voit.*	L'enfant salut toujours les grandes personnes de la communauté quand il se rencontre avec eux, même ceux qui sont dans sa maison et ceux qui sont dehors	L'enfant salut parfois les grandes personnes même ceux qui sont dans sa maison et de la communauté, et parfois ne les salut pas.	L'enfant refuse de saluer les grandes personnes de la communauté.
**6. À tie kɛ̀ koboo wó à yi tɛ́ pe e mɛɛ̀ tima nɛ̀, nye kɛ̀ éà mɛɛ̀ nɛ̀ ?**	**Yandɛ é à mɛɛ̀ èe kɔ̀pɛ kɛ̀** kobo na gbao w**ó à manà pɛ́ nɛ̀ .**	L'enfant fait toujours les commissions sans refuser.	L'enfant fait parfois les commissions, mais parfois il peut	L'enfant refuse de faire ce qu'on lui dit.

Question de réflexion	Comportement cible/attendu	Maitrisé (Le comportement est bien, n'est pas problématique)	En cours de maitrisé (Parfois bien, mais un peu problématique)	Non maitrisé (Le comportement est souvent problématique)
**Commissions.** *Quand une grande personne lui demande de faire une commission, qu'est-ce qu'il fait ?*	*L'enfant fait tous ce que les grandes personnes du village lui disent.*		hésiter, faire ça lentement, ou va vouloir finir de jouer d'abord.	
**7. A tie kɛ̀** kobo na gbao **wó à tɔ pɛ́ tɛ́ lɛ́wu nɛ̀ nye Kɛ éà mɛɛ̀ nɛ̀ ?**<br>**Ecouter.** *Quand une grande personne de la communauté lui donne des conseils, qu'est-ce qu'il fait ?*	**Yandɛ bà jɛ lewù kɛ̀o** kobo na gbao **wó à manà pɛ́ nɛ̀.**<br>*L'enfant écoute les conseils des grandes personnes de la communauté*	L'enfant écoute attentivement des grandes personnes du village, et pose des questions pour mieux comprendre, et aime rester avec les grandes personnes.	L'enfant fait la confusion entre les bons et les mauvais conseils. Il écoute parfois les conseils de ses amies, et parfois les conseils des grandes personnes de la communauté.	L'enfant ne suit pas les conseils des grandes personnes du village. Il s'avance sans écouter. Il prend la parole sans qu'on lui donne, et simplifie les choses que les grandes personnes lui disent.
8.Tɛ́ pe é à mbɛ̀ bɛlà à ngɛ̀ɛ́ó ndé ná dotò yékeyèkè ?<br>**Qualité.** *Est-ce qu'il finit ses travaux sans se distraire ?*	**Yandɛ bà mɛɛ̀ bèlà á ngɛ́ jókò ndé ná dotò yékeyèkè ?**<br>*L'enfant fait bien son travail sans distraction.*	L'enfant qui est concentré quand il travail. Il ne joue pas pendant qu'il travail. L'enfant peut mener une tache appropriée (ex : laver les assiettes, dessiner une fleure) du début jusqu'à la fin sans se distraire.	L'enfant est engagé dans son travail, mais il aime aussi les jeux. Parfois il laisse son travail pour jouer, mais il a la volonté de faire son travail. L'enfant peut se concentrer sur certaines tâches appropriés (ex : laver les assiettes, dessiner la fleure) mais pas d'autres tâches appropriées.	L'enfant fait du désordre quand il travail. Il ne reste pas concentré sur son travail. Il part souvent jouer, même sans finir son travail. L'enfant est facilement distrait quand il travail, ne finit pas souvent son travail sans l'appui d'une autre personne.
**9 et 10. Bà ngomà pe**è wá tie kpódé kɛ̀ nɔɔ̀ Yandɛ é geè ngí a lu tɛ́ kɔlɛ́ nɛ. E mɛɛ̀ ngi pe ?<br>**Agir.** *Parle-moi d'un temps qu'un autre enfant voulait bagarrer avec lui. Comment a-t-il réagi ?*	**9. Pe boo wá dɔ tɛ we, Yandɛ ndé na gelo.** *Si les gens viennent avec les problèmes, l'enfant ne réagit pas.*	L'enfant réagit pour se défendre, mais ne s'engage pas dans la bagarre. S'il fait du mal à l'autre, il se sent coupable. Il n'a pas des habitudes violentes, il peut être fâché mais n'engage pas la bagarre.	L'enfant réagit excessivement pour se défendre, et peut parfois s'engager dans la bagarre. S'il fait du mal à l'autre, il se sent parfois coupable.	L'enfant agit sans se rendre compte de ses actions. Il commence directement le bagarre si quelqu'un le menace, sans réfléchir ou hésiter.
	10. **Yandɛ é mɛɛ́ kɔlɛ tɛ biɛ̀ lɛ̀ Yandɛo ode**. *L'enfant ne bagarre pas avec les autres.*	L'enfant bagarre ou tape sur les autres enfants s'il est provoqué par les autres, mais n'incite pas le bagarre	L'enfant peut parfois provoquer la bagarre, et tape sur les autres enfants s'il est provoqué par les autres.	L'enfant bagarre souvent avec les autres, il provoque les autres enfants
**11. A tie kɛ̀ é à jɛ̀ tɛ́ kábu nɛ̀ tɛ́ pe é à bɛ̀ tɛ kabu a ngɛ yà** ?	**Yandɛ tɛ na njiì a kɛ na jɛ̀ kabu ndé pe kobo à yangɛ́**	L'enfant peut se fâcher, mais pas tellement et pas chaque jour. Il est facile de le consoler, ou il se	L'enfant peut se fâcher, parfois tellement, mais pas chaque jour. Il	L'enfant se fâche souvent, ne se calme pas facilement, même avec l'appui d'un adulte

Question de réflexion	Comportement cible/attendu	Maitrisé (Le comportement est bien, n'est pas problématique)	En cours de maitrisé (Parfois bien, mais un peu problématique)	Non maitrisé (Le comportement est souvent problématique)
**Calmer**. *Quand il se fâche, est-ce qu'il se calme facilement ?*	*L'enfant peut se calmer après qu'il/elle se fâche, sans l'aide d'un adulte*	calme sans qu'une grande personne le console.	se calme quand les grandes personnes le console.	
**12. Nye kɛ̀ é à mɛɛ̀ pe à mɛlɛ̀** siti tɛ́ bo nɛ̀ ?<br>**Pardonner**. *Que fait-il quand il fait du mal à quelqu'un par accident ?*	**Éà gbɔ bibi tɛ bo à tie kɛ̀ a mɛɛ̀lɛ̀ sítí tɛ bo nɛ̀.** *L'enfant demande pardonne après avoir fait du mal à quelqu'un.*	S'il fait du mal a quelqu'un par accident, l'enfant demande pardon. Il peut continuer à jouer avec l'autre après l'incident.	L'enfant peut demander pardon parfois, mais pas chaque fois qu'il fait du mal à quelqu'un. Si une grande personne lui dit de demander pardon, il le fait.	Il ne demande pas pardon après avoir fait du mal à un autre enfant par accident. Il n'est pas conscient de ce qu'il a fait.
**13. ngomà pée èé kɛ̀ éà mɛɛ̀ à tie kɛ é à ye tɛ́ èe a nɔɔ̀ bó nɛ ?**<br>**Arracher**. *Parle-moi de ce qu'il fait quand il veut quelque chose qui appartient à autrui.*	**Yandɛ é gboò éeo ode.** *L'enfant n'arrache pas des choses*	L'enfant demande toujours avant de prendre quelque chose d'autrui. Il n'arrache presque jamais.	L'enfant demande parfois des choses d'autrui. Parfois il ne le fait pas, il prend sans demander, surtout quand il veut la chose tellement (ex : quand il a bien faim)	L'enfant arrache souvent les choses des autres, sans demander.
**14. ngomà peè èe kɛ̀ é à mɛɛ̀ à tie kɛ̀ nɔɔ̀ bo é à ye tɛ yangà ágɛ́ nɛ̀ ?**<br>**Aider.** *Parle-moi de ce qu'il fait quand quelqu'un a besoin d'aide.*	**Yandɛ bà yangà boo a bèlà a ngó.** *L'enfant aide les autres en besoin.*	L'enfant réagit immédiatement quand il constat qu'une personne est en difficulté. Il aide les autres, même souvent sans qu'on lui demande.	L'enfant aide ses amies d'abord, mais n'aide pas souvent les autres. L'enfant aide les autres si tu le demande, mais ne le fait pas souvent sans qu'on lui demande.	L'enfant refuse d'aider les autres, même si on lui demande. Il priorise les jeux, et n'a pas l'habitude de venir en appui aux autres.
**15. Éà mɛ́ɛ̀ pe à tie Kɛ̀ lotì a ngɛ́ é tɛ a yùwà nɛ̀ ?**<br>**Compatir.** *Qu'est-ce qu'il fait si son ami est triste ?*	**Yandɛ é tɛ bumɛ na yuwà. éà jɛ̀ pe na biyɛ́kɛ̀ bíɛ̀ bo bà jɛ̀ nɛ̀.** *L'enfant a un cœur de pitié. Il comprend comment les autres se sentent.*	L'enfant partage facilement le chagrin de l'autre. Si une autre personne est fâchée ou triste, l'enfant veut le consoler.	L'enfant observe les autres mais ne réagit pas pour montrer sa sympathie. Par exemple, si quelqu'un pleure, l'enfant ne le console pas et ne pleure pas, mais il voit la personne en état de tristesse.	L'enfant ne comprend pas les sentiments des autres. L'enfant ne réagit pas si quelqu'un est triste ou fâché. Il peut se moquer de la personne au lieu de sympathiser avec eux.
**16**. **É a mɛɛ̀ pe à tie na mɛɛ̀ nɔɔ̀ èe ngbenyi bèlà kɛ̀ é mɛlɛ so nɛ?**<br>***Tenter*** *: Comment agit-il face à une nouvelle tache ou activité qu'il n'a jamais fait ?*	**Yandɛ bà lɛ́ bèlà ná tɔtɔo ndé na jɛ̀ gò.** *L'enfant tente des nouvelles activités sans peur.*	L'enfant fait ce qu'on lui montre. Il a la volonté d'essayer, et il a confiance dans ses capacités. Ex : si tu lui demande de grimper un nouvel arbre ou écrire une nouvelle lettre, il essai de le faire petit à petit.	L'enfant est un peu réticent pour essayer des nouvelles activités. Il observe les autres avant de tenter lui-même. Il y a certaines nouvelles activités qu'il fait sans peur, mais d'autres qu'il ne tente pas.	L'enfant a peur de tenter des nouvelles activités. L'enfant n'a pas confiance dans ses capacités. Il refuse d'essayer des nouvelles activités. Ex : si on lui demande d'écrire une nouvelle lettre ou de grimper un nouvel arbre, il refuse

Question de réflexion	Comportement cible/attendu	Maitrisé (Le comportement est bien, n'est pas problématique)	En cours de maitrisé (Parfois bien, mais un peu problématique)	Non maitrisé (Le comportement est souvent problématique)
**17 (parents uniquement) A tie kɛ̀ é à nyɛ̀ tɛ ngo nɛ̀, tɛ é à ye pe nɔɔ̀ boo e yangɛ́ ?**<br>***Se laver.*** *Quand il/elle se lave, est-ce qu'il/elle veut que tu l'aide ?*	**Yàndɛ bà ye na nyɛ̀ ngo nde na ye yanga koboo (a bo nɔɔ̀ tie e tɛ a ye pe wó yangɛ́).**<br>*L'enfant veut se laver sans l'aide de ses parents (mais peut parfois avoir besoin d'appui).*	L'enfant essaie de se laver, sans l'aide de son parent, mais peut parfois avoir besoin d'appui.	L'enfant essaie parfois de se laver, mais demande souvent l'aide de ses parents.	L'enfant ne veut pas se laver indépendamment (et/ou a besoin de beaucoup d'appui) de sonparent.
**18 (parents uniquement) Tɛ pe là a mò bà mɛɛ̀ bèlà a nda tie kɔ̀pɛ̀ ?**<br>***Travailler****. Est-ce que ton enfant fait des travaux régulièrement à la maison*	**É à yangà boo a ngɛ́ó a bèlà nà ndao tɛ nɔɔ̀ lɛ̀ bèlào.**<br>*Il aide les gens dans les travaux de la maison et bien d'autres.*	L'enfant est toujours disponible à travailler. Il fait ses travaux sans qu'on lui ne demande/envoi.	Parfois l'enfant va privilégier les jeux avant de faire ses travaux, il peut oublier les travaux, mais il fait si tu le rappelle.	L'enfant veut seulement jouer, il ne fait pas des travaux
